# Supplementary material for: Effect of a pay-it-forward strategy on reducing HPV vaccine delay and increasing uptake among 15- to 18-year-old girls in China: A randomized controlled trial
Source: PLoS Med. 2025 Jul 31;22(7):e1004535. doi: 10.1371/journal.pmed.1004535 (PMC12331080; doi:10.1371/journal.pmed.1004535)
Supplement: S2 Checklist — (DOCX) [file pmed.1004535.s002.docx]

# CONSERVE Checklists

Use CONSERVE-CONSORT for completed trial reports and CONSERVE-SPIRIT for trial protocols.

| CONSERVE-CONSORT Extension: [DATE] | | | | | |
| --- | --- | --- | --- | --- | --- |
| Item | Item Title | Description | | | Page No. |
| I. | Extenuating Circumstances | Describe the circumstances and how they constitute extenuating circumstances. | | | Second paragraph of Participant recruitment and screening |
| II. | Important Modifications | 1. Describe how the modifications are important modifications. | | | Second paragraph of Participant recruitment and screening |
|  |  | 1. Describe the impacts and mitigating strategies, including their rationale and implications for the trial. | | | (see below) |
|  |  | 1. Provide a modification timeline. | | | Second paragraph of Participant recruitment and screening, Fig B in S1 File |
| III. | Responsible Parties | State who planned, reviewed and approved the modifications. | | | Second paragraph of Participant recruitment and screening |
| IV. | Interim data | If modifications were informed by trial data, describe how the interim data were used, including whether they were examined by study group, and whether the individuals reviewing the data were blinded to the treatment allocation. | | | NA |
| CONSORT Number and Item | | For each row, if important modifications occurred check “direct impact” and/or “mitigating strategy” and describe the changes in the trial manuscript or supplement. Check “no change” for items that are unaffected in the extenuating circumstance. | | | Page No. |
|  |  | No Change | Impact* | Mitigating Strategy** |  |
| 1 | Title and abstract | X |  |  |  |
| 2 | Introduction | X |  |  |  |
| 3 | Methods: Trial Design | X |  |  |  |
| 4 | Methods: Participants | X |  |  |  |
| 5 | Methods: Interventions | X |  |  |  |
| 6 | Methods: Outcomes | X |  |  |  |
| 7 | Methods: Sample Size | X |  |  |  |
| 8-10 | Methods: Randomisation | X |  |  |  |
| 11 | Methods: Blinding | X |  |  |  |
| 12 | Methods: Statistical methods | X |  |  |  |
| 13 | Results: Participant flow | X |  |  |  |
| 14 | Results: Recruitment |  | X |  | Second paragraph of Participant recruitment and screening |
| 15 | Results: Baseline data | X |  |  |  |
| 16 | Results: Numbers analysed | X |  |  |  |
| 17 | Results: Outcomes and estimation | X |  |  |  |
| 18 | Results: Ancillary analyses | X |  |  |  |
| 19 | Results: Harms | X |  |  |  |
| 20 | Discussion: Limitations |  | X |  | Fifth paragraph of Discussion |
| 21 | Discussion: Generalisability | X |  |  |  |
| 22 | Other information: Registration | X |  |  |  |
| 23 | Other information: Protocol | X |  |  |  |
| 24 | Other information: Funding | X |  |  |  |
| *Aspects of the trial that are directly affected or changed by the extenuating circumstance and are not under the control of investigators, sponsor or funder.  **Aspects of the trial that are modified by the study investigators, sponsor or funder to respond to the extenuating circumstance or manage the direct impacts on the trial. | | | | | |
