# Supplementary material for: Effect of a pay-it-forward strategy on reducing HPV vaccine delay and increasing uptake among 15- to 18-year-old girls in China: A randomized controlled trial
Source: PLoS Med. 2025 Jul 31;22(7):e1004535. doi: 10.1371/journal.pmed.1004535 (PMC12331080; doi:10.1371/journal.pmed.1004535)
Supplement: S1 File — Fig A. Overview of pay-it-forward. Fig B. Study site recruitment over time. Fig C. Community engaged postcards. Fig D. Educational pamphlet. Fig E. Percentages of donations of vaccine costs by study sites. Fig F. Breakdown of financial costs by category in proportions in the two arms. Fig G: Translated example hand-written postcard messages from the participants. Appendix A. Telephone recruitment script. Appendix B. Community engagement activities. Appendix C. Questionnaire and codebook. Appendix D. Data analysis code. Appendix E. Video abstract. Table A. Cost calculations (in 2022 USD) for the study. Table B. Recruitment, appointment, and reasons for non-presence for vaccination. (DOCX) [file pmed.1004535.s004.docx]

Pay‑it‑forward strategy reduced HPV vaccine delay and increased uptake among catch-up age girls: A randomized clinical trial

**S1 Supporting information**

[Figure A. Overview of pay-it-forward 2](#_Toc197958280)

[Figure B. Study site recruitment over time 2](#_Toc197958281)

[Figure C. Community engaged postcards. 2](#_Toc197958282)

[Figure D. Educational pamphlet 3](#_Toc197958283)

[Figure E. Percentages of donations of vaccine costs by study sites 8](#_Toc197958284)

[Figure F. Breakdown of financial costs by category in proportions in the two arms 8](#_Toc197958285)

[Figure G: Translated example hand-written postcard messages from the participants 9](#_Toc197958286)

[Appendix A. Telephone recruitment script 9](#_Toc197958287)

[Appendix B. Community engagement activities 10](#_Toc197958288)

[Appendix C. Questionnaire and codebook 10](#_Toc197958289)

[Appendix D. Data analysis code(R·version·4.2.1) 13](#_Toc197958290)

[Appendix E. Video abstract 23](#_Toc197958291)

[Table A. Cost calculations (in 2022 USD) for the study 23](#_Toc197958292)

[Table B. Recruitment, appointment, and reasons for non-presence for vaccination 24](#_Toc197958293)

# Figure A. Overview of pay-it-forward


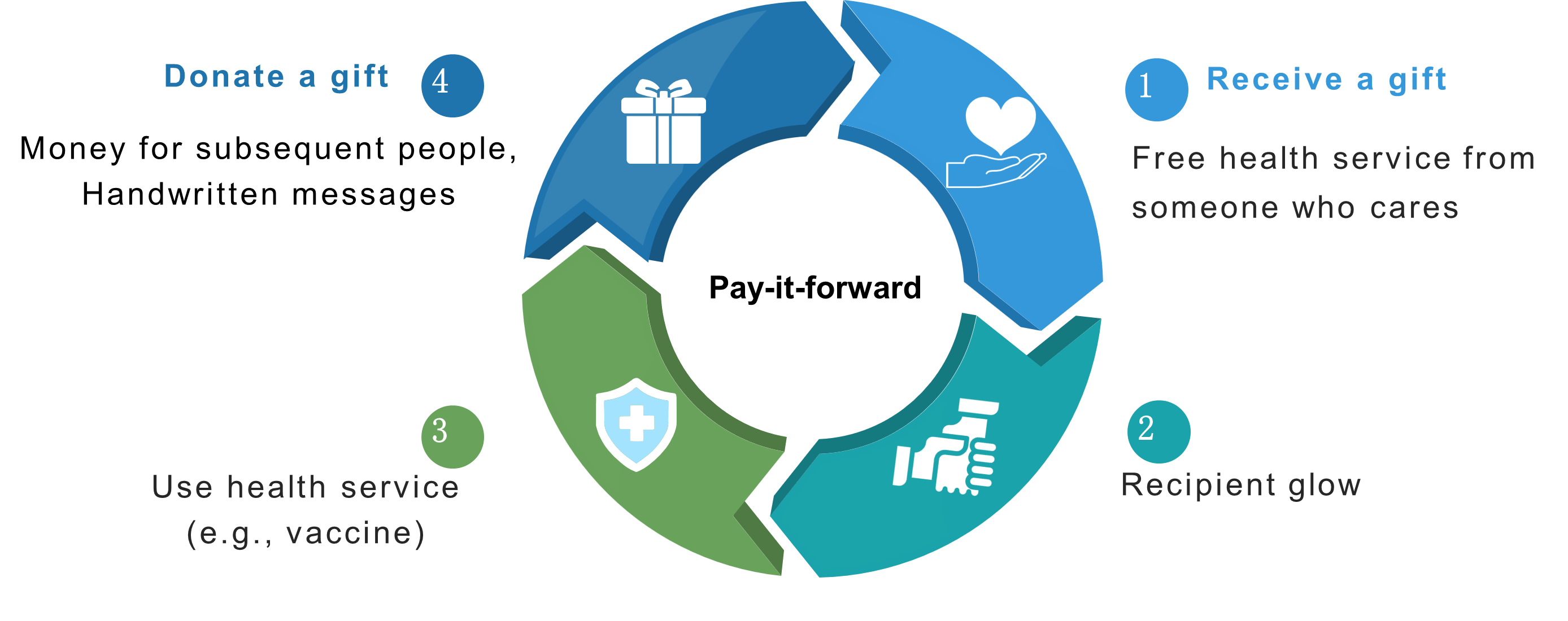


# Figure B. Study site recruitment over time


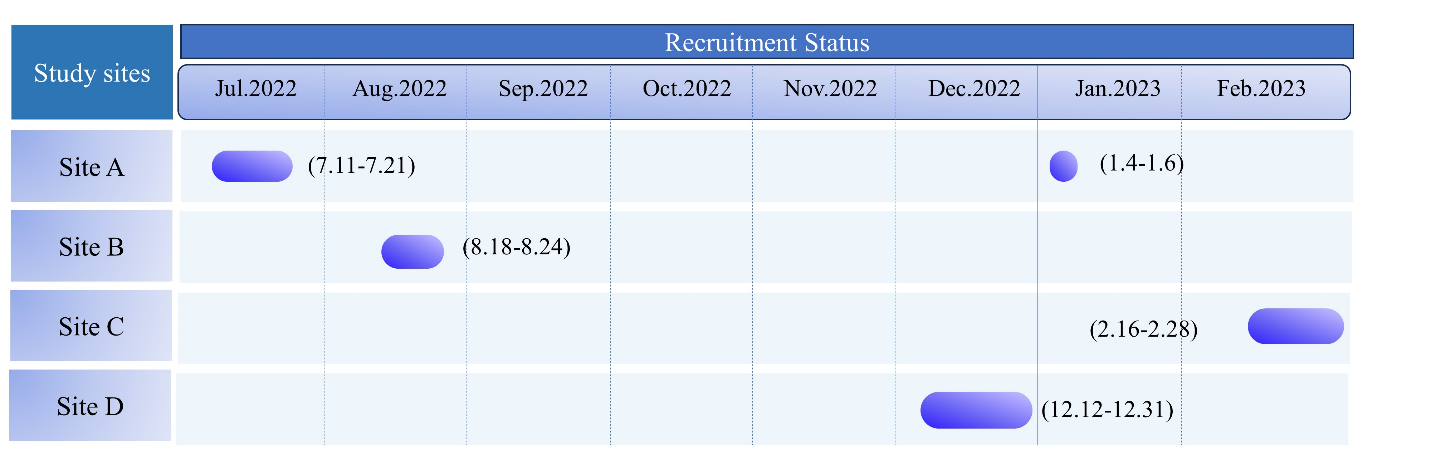


# Figure C. Community engaged postcards.

| 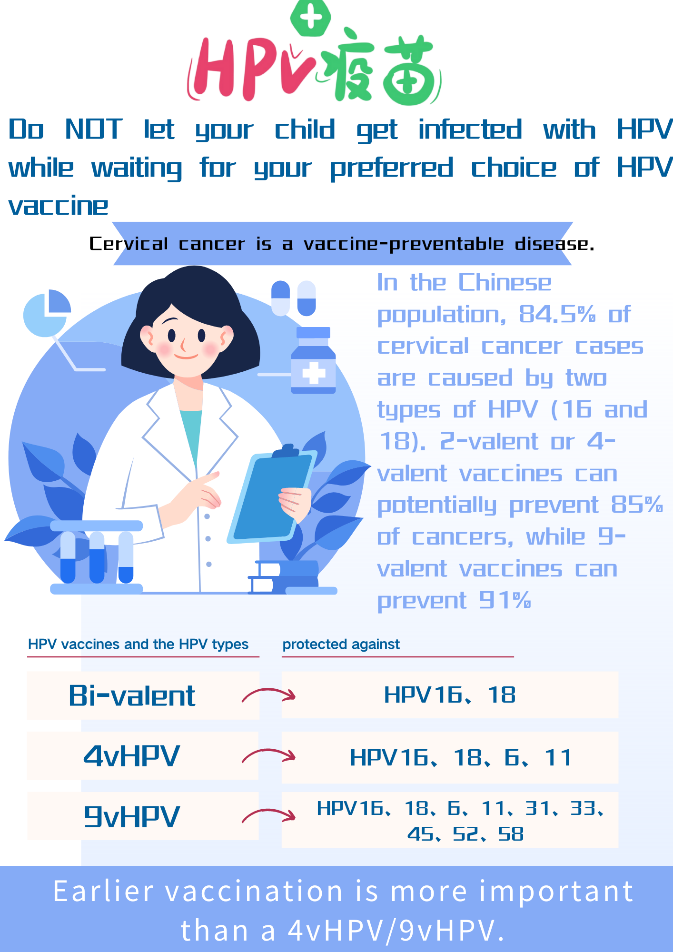Reference: Chen W, Zhang X, Molijn A, Jenkins D, Shi JF, Quint W, Schmidt JE, Wang P, Liu YL, Li LK, Shi H, Liu JH, Xie X, Niyazi M, Yang P, Wei LH, Li LY, Li J, Liu JF, Zhou Q, Hong Y, Li L, Li Q, Zhou HL, Bian ML, Chen J, Qiao YL, Smith JS. Human papillomavirus type-distribution in cervical cancer in China: the importance of HPV 16 and 18. Cancer Causes Control. 2009 Nov;20(9):1705-13. doi: 10.1007/s10552-009-9422-z. Epub 2009 Aug 25. PMID: 19705288. |
| --- |
| 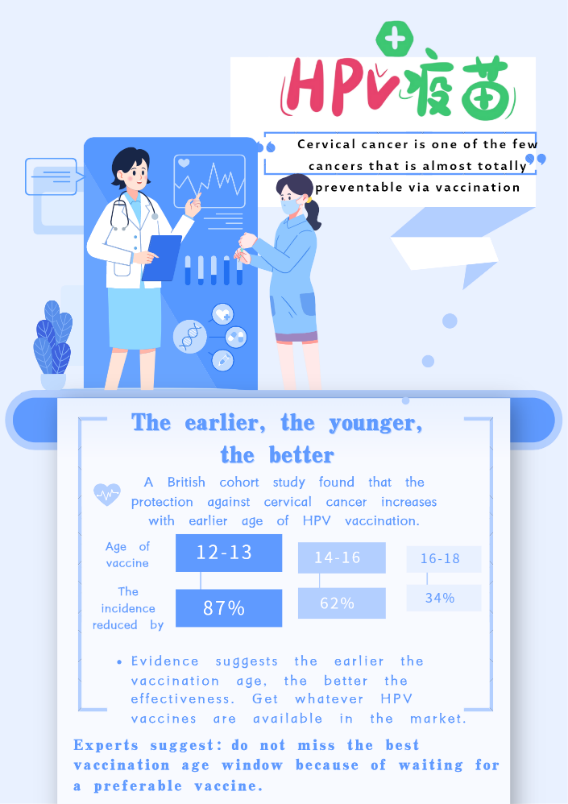[1] Reference: Falcaro M, Castañon A, Ndlela B, Checchi M, Soldan K, Lopez-Bernal J, et al. The effects of the national HPV vaccination programme in England, UK, on cervical cancer and grade 3 cervical intraepithelial neoplasia incidence: a register-based observational study. Lancet. 2021;398(10316):2084–92. Available from: https://doi.org/10.1016/S0140-6736(21)02178-4 |
|  |
| 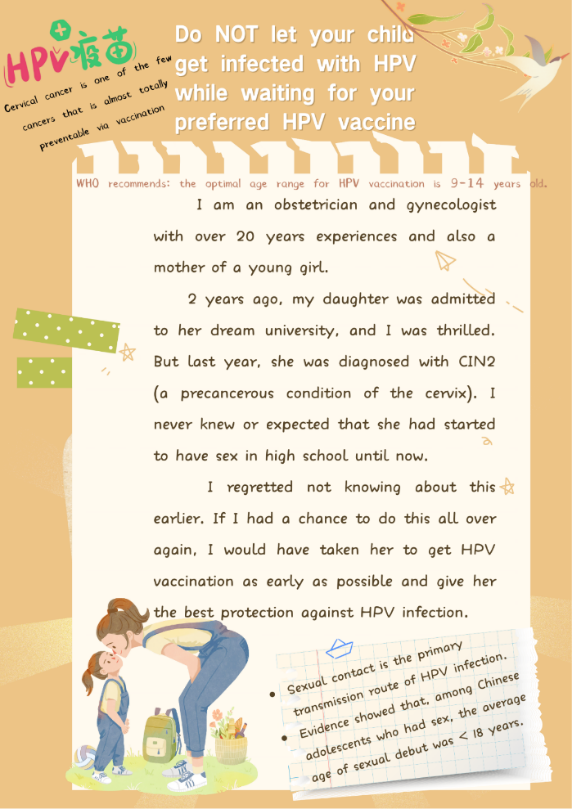[2] Reference: Yang J, Shen M, Wang Z, et al. Prevalence and influencing factors of sexual behavior among university freshmen. Chinese Journal Public Health 2021; 37(03): 431-4. |


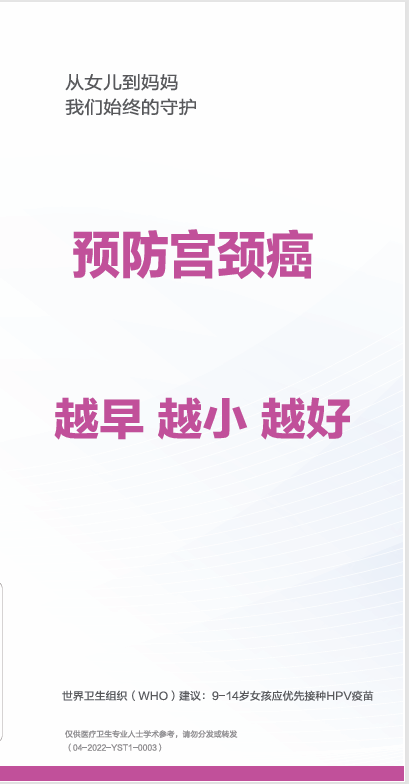


# **Figure D. Educational pamphle**t

We protect daughters and mothers

Cervical cancer prevention

The earlier, the younger, the better

WHO recommends primary target population for HPV vaccination is adolescent girls aged 9–14 years for preventing cervical cancer


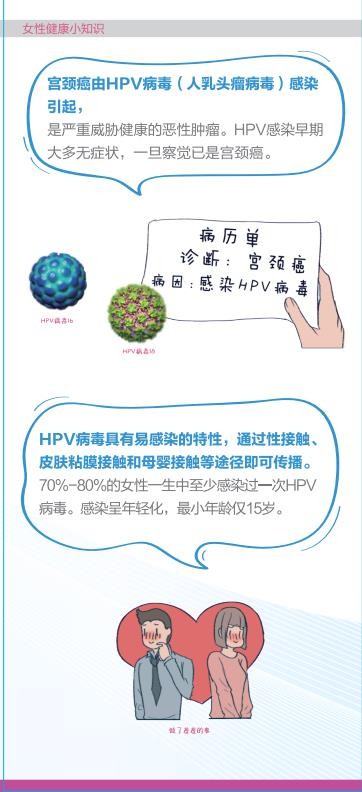


Persistent infection with high-risk HPV types can lead to cervical cancer, which is a serious threat to health. HPV infections are mostly asymptomatic in the early stage, but if left undetected, there is a high risk of progression.

HPV viruses are spread through contact with infected genital skin, mucous membranes, or bodily fluids, and can be transmitted through sexual intercourse and vertical transmission of HPV from mother

to child.

About 70-80% of women have been infected with HPV at least once in their lives. Infection tends to occur at a younger age, with the youngest being 15 years old.


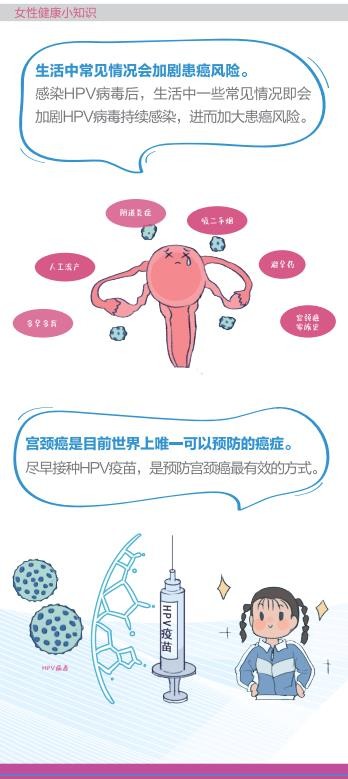


After acquiring HPV viruses, common factors in daily life may become risk factor for persistent infection with high-risk HPV types, contributing to progression to cervical cancer:

These factors can be multiple full-term pregnancies, early first sexual intercourse, history of STI infection, smoking or breathing in secondhand smoke, long-term use of oral contraceptives, family history of cervical cancer etc.

Cervical cancer is a vaccine-preventable disease.

Early vaccination against HPV is the most effective way

to prevent cervical cancer.


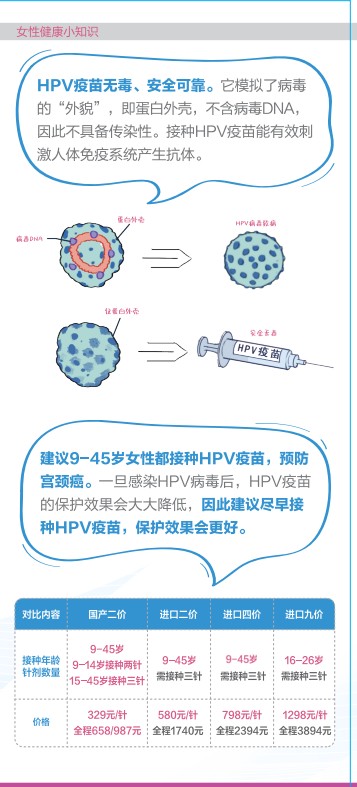


HPV vaccination is a safe and effective way to help prevent cervical cancer. The current HPV vaccines are based on virus- like particles (VLPs) that are formed by HPV surface components. VLPs are not infectious because they lack the virus’s DNA. The HPV vaccines effectively stimulate the body’s immune system to produce antibodies.

The HPV vaccine is recommended for females aged 9 to 45 years to prevent cervical cancer. Once infected with HPV viruses, HPV vaccination has less benefits. It is recommended to get the HPV vaccine as early as possible for better protection.

| **Comparison Content** | **Domestic bivalent vaccine**  **(Cecolin)** | **Imported bivalent vaccine (Cervarix)** | **Imported quadrivalent vaccine (Gardasil)** | **Imported 9-valent HPV vaccine (Gardasil 9)** |
| --- | --- | --- | --- | --- |
| Age for Vaccination and Doses Needed | 9-45 years old  A 2-dose schedule is recommended for age 9- 14 and 3 doses are recommended for age 15- 45 | 9-45 years old A 3-dose schedule is recommended for age 9-45 | 9-45 years old A 3-dose schedule is recommended for age 9-45 | 16-26 years old (this age range was recently amended to 9-45 years in August 2022)  A 3-dose schedule is recommended for age 9-45 |
| Price | 329 yuan/dose (US$49.78 at the exchange rate of US$1=6.61CNY)  658/987 yuan altogether (US$99.56/US$1 49.34) | 560 yuan/dose (US$84.72)  1740 yuan altogether (US$2 54.16) | 798 yuan/dose (US$120.72)  2394 yuan altogether (US$362.18) | 1298 yuan/dose (US$196.37)  3894 yuan altogether (US$580.11) |

Women’s health tips


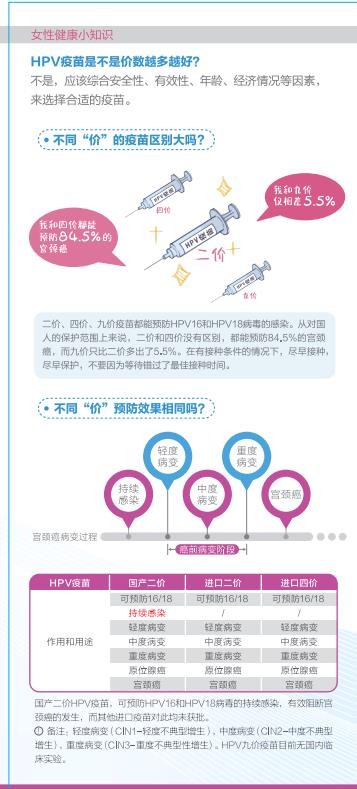


- Is the 9-valent HPV vaccine the best？

No. The choice of HPV vaccine should be based on a number of factors, including

safety, efficacy, age, price and so on.

- Are there any differences between these HPV vaccines？

Bivalent or quadrivalent HPV vaccines can potentially reduce cervical cancer by 84.5%, which is only 5.5% less than the 9-valent HPV vaccine.

Current evidence suggests that licensed HPV vaccines have relatively similar effectiveness in preventing cervical cancer. With regards to cervical cancer prevention, all licensed HPV vaccines provide high protection against HPV-16 and HPV-18 which are associated with 84.5% of cervical cancer cases in China. It is recommended to vaccinate girls as early as possible, and don’t miss the best vaccination time due to waiting.

- How effective are HPV vaccines?

| **HPV vaccine** | **Domestic bivalent**  **vaccine** | **Imported bivalent**  **vaccine** | **Imported**  **quadrivalent vaccines** |
| --- | --- | --- | --- |
| **Functions and Uses** | prevent HPV16/18 | prevent HPV16/18 | prevent HPV16/18 |
|  | Persistent HPV  infection | / | / |
|  | Mild lesions | Mild lesions | Mild lesions |
|  | Moderate lesions | Moderate lesions | Moderate lesions |
|  | Severe lesions | Severe lesions | Severe lesions |
|  | In situ  adenocarcinoma | In situ  adenocarcinoma | In situ  adenocarcinoma |
|  | cervical cancer | cervical cancer | cervical cancer |

The domestic bivalent HPV vaccine can prevent persistent infection of HPV-16 and HPV-18 viruses and effectively prevent cervical cancer.

①Note: mild precancerous lesions(CIN1-mild dysplasia),moderate precancerous lesions(CIN2-

moderate to marked dysplasia),severe precancerous lesions(CIN3-severe dysplasia to carcinoma in situ). There are currently no clinical trials for domestic 9- valent HPV vaccines.

# Figure E. Percentages of donations of vaccine costs by study sites

# Figure F. Breakdown of financial costs by category in proportions in the two arms

# Figure G: Translated example hand-written postcard messages from the participants

| 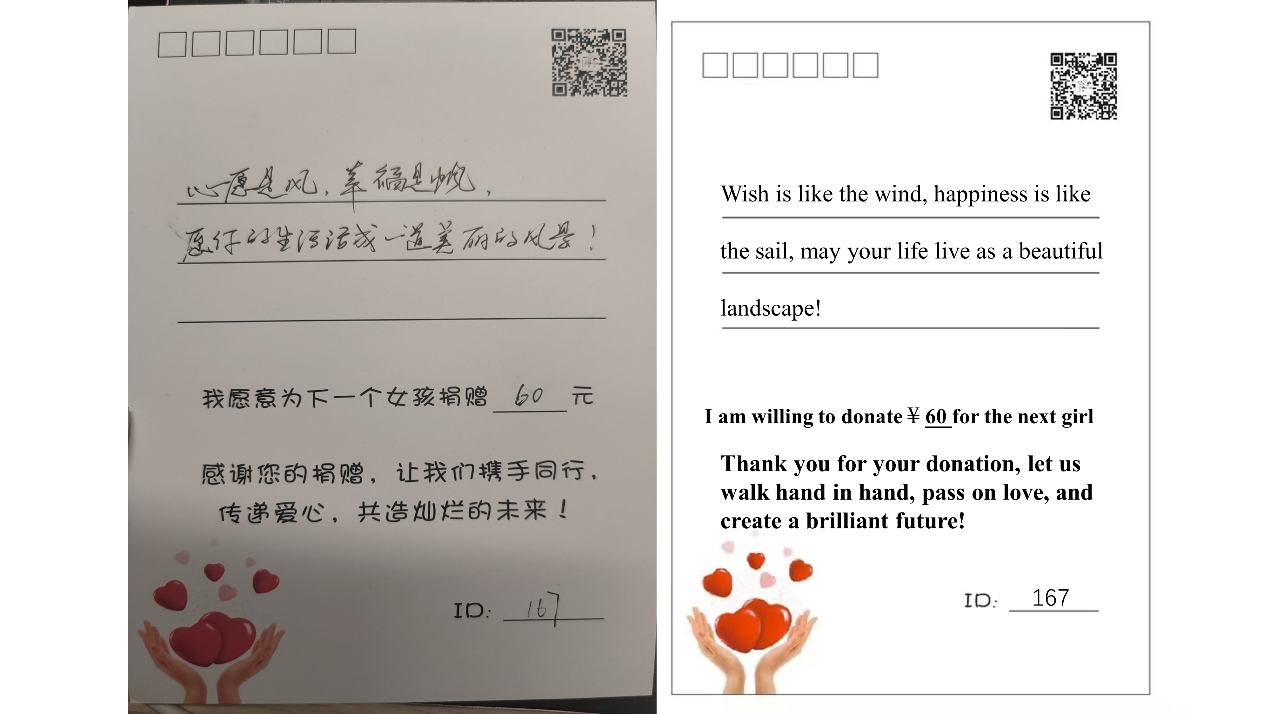 |
| --- |

# Appendix A. Telephone recruitment script

**Note: Individuals are generally busy or reluctant to talk too much to a person calling from an unfamiliar phone number, the purpose of the telephone recruitment is to spur interests and inviting participants who have a general interest in HPV vaccination to the clinic for a more thorough introduction to the project and consenting process. For uncontactable participants after three attempts, the status was confirmed as uncontactable.**

**Hello, madam/sir, I am a staff member of the XX Community Health Service Center. May I ask if you are the parent of XXX? I would like to invite you to participate an HPV vaccination program jointly organized by our center and School of Public Health of Sichuan University. It takes a few minutes only **[if a parent does not hang up, continue asking one quick question on HPV vaccination history; if they hang up, end of recruitment process]**.

1) Has your girl received the cervical cancer vaccine/HPV vaccine**? [If no, continue introducing the project; if yes, end of recruitment process]**

2) Our project aims to provide cervical cancer vaccinations for girls aged 15-18. By participating in this project, you will have an opportunity to receive a cash subsidy for your girl's HPV vaccination (50% chance). And when you visit us in person, you will have an opportunity to receive a lottery for winning a quota for a quadrivalent/nonavalent vaccine (5% chance). Are you interested in hearing more about this? **[If yes, continue introducing the project; if no, end of recruitment process]**.

3) Cervical cancer is a type of cancer caused by HPV viruses, and HPV vaccine can effectively prevent cervical cancer. The Chengdu municipal government has rolled out a subsidized HPV vaccination program for girls between the ages of 13-14 years old only, and our project aims to cover girls between the ages of 15-18 years old. Your girl is eligible for HPV vaccination through our project and she is lucky to be selected out of >1000 girls living in the neighborhood. If you are willing to participate, you only need to visit our clinic located at [provide address] at any time between 9am-4pm during [period of recruitment]. We will provide more detailed materials and information about the project when you visit us. You will also receive a CNY50 as a compensation for your transportation. Note that anyone who come in person and participate an online questionnaire has a 50% chance to win a CNY330 for supporting HPV vaccination. Would you like us to book a time slot for you now? [If yes, book a time slot and send the location via a text message; if no, continue to the next question.]

4) Can I ask why you are not interested in this project? [Write down main reasons for declining to participate; if they suggested they need some time to consider or discuss with family, then offer to follow up with another telephone call].

# Appendix B. Community engagement activities

**Part A: Stakeholder engagement meetings**

We identified a locally influential public health practitioner (i.e., the head of the vaccination clinic at Site A) as a key person for local resources coordination and understanding contextual backgrounds. Between 15 April 2021 and 30 Jun 2022 before we started data collection, we had 18 meetings (online and in-person) with the practitioner and his team to iteratively refine the trial design. After completing the pilot study (January 2022 to February 2022 with 100 eligible girls), we organized a multi-stakeholder advisory meeting on 22 April 2022 to discuss the pilot results and refinement plans for the trial. The multi-stakeholder panel included an HPV vaccination policymaker from Chengdu municipal health bureau, two public health practitioners in women and child health, two vaccine research experts, two global health researchers, and two infectious diseases researchers. More details can be found in the protocol.

**Part B: Launch meetings**

We organized a pilot launch meeting and a trial launch meeting respectively on 30 December 2021 and 15 June 2022. The main purposes of the two launch meetings were to engage key stakeholder representatives, build local capacity and momentums for the project. Six key collaborative persons from the four community health centers, the research team, a vaccine expert from the Gates Foundation, two parent representatives (a father and a mother), and supporting staff including an organizational leader from Site A joined the launch meetings. We presented the overall project design and interventional materials, clarified the objectives, plans, and procedures, and responded to all questions attendees had. We made amendments based on stakeholder feedback before data collection started in July 2022. We released newsletters via official WeChat accounts or websites of the four community health centers to publicize the launch meetings to gain public trust in our project.

**Part C: Qualitative interviews with participating caregivers**

We conducted four focus group discussions (21 caregivers) between June to August 2022 to understand parental decision for their daughter’s HPV vaccination. We identified potential factors contributing to Chinese caregivers’ intention to delay their daughters’ HPV vaccination over receiving the best available HPV vaccines. In summary, these included concerns about vaccine safety, knowledge gaps and misinformation, perceived sexual inactivity of adolescents, preference for 9vHPV vaccine and vaccine shortage, and inadequate health communication. More details can be found in our published paper [here](https://bmcpublichealth.biomedcentral.com/counter/pdf/10.1186/s12889-024-17697-6.pdf).

**Part D: Community engaged postcard messages**

Participants were engaged in two ways in the postcard message generation. First, during the intervention design process, the postcard covers were co-designed by university college students. The cover messages were designed based on the focus group discussion findings and aim to address key knowledge gaps identified (see Appendix Figure 2). Specifically, these cover messages focused on: a) how effective of different types of HPV vaccines are in preventing cervical cancer, b) why it is optimal to vaccinate an eligible girl at an earlier age rather than later, and c) parental perception of age of sexual debut of their daughter. Second, during the project implementation process, participating caregivers and girls were invited to write a postcard message to support other girls (see Appendix 6).

**Part E: Receiving and giving**

This is a unique way of engaging participants by giving them a subsidized service and then offering them to an opportunity to give back to the community via leveraging human kindness and cultivating community solidarity. We informed participants that the subsidy of 330 RMB was contributed by generous individuals. In the event of being willing to receive the subsidy and vaccinate, participants were then offered an opportunity to voluntarily donate to support other girls. Existing data showed that the process may create a sense of belonging and people who received help are more likely to help others in return.

# Appendix C. Questionnaire and codebook

| **VARIABLE NAME** | **MEANING** | **VALUE** |
| --- | --- | --- |
| arm | group | 0=standard-of-care arm  1=pay-it-forward arm |
| study site | community health centers | 1=Yulin Community Health Service Center (site A)  2=Longtan Community Health Service Center (site B)  3=Xinjin District Maternal and Child Healthcare Hospital (site D)  4=Third People’s Hospital of Chengdu Eastern New Area (Site C) |
| appointment | Whether to make an appointment for vaccination | 0=No  1=Yes |
| uptake | Whether receive the first dose of HPV vaccine | 0=No  1=Yes |
| uptake2 | Whether receive the second dose of HPV vaccine | 0=No  1=Yes |
| donation | The actual donation amount **(RMB)** | 1USD=6.89RMB  (Only the participants who receive the HPV vaccine in the pay-it-forward arm have the data on this item) |
| postcard writing | Whether write a postcard for the future girls | 0=No  1=Yes  (Only the participants who receive the HPV vaccine in the pay-it-forward arm have the data on this item) |
| **PART A BASIC INFORMATION** | | |
| A0.1 | The date of filling the questionnaire | YY-MM-DD |
| Age1 | Age of girls | years |
| Age2 | Age of caregivers | years |
| A1 | Sex of the participating caregiver | 1=male  2=female |
| A2 | Ethnicity | 1=Han  2=Others |
| A4 | Marital status of the caregivers | 1=unmarried  2=engaged or married  3=separated or divorced  4=widowed  5=others |
| A6 | The highest educational level of participating caregiver | 1. Primary school or below 2. Junior middle school 3. High school 4. Undergraduate or college 5. Postgraduate |
| A7 | The current occupation of participating caregiver | 1. Civil servant 2. Farmer 3. Ordinary workers (blue collar or labor-intensive work) 4. Company staff (white collar or office work) 5. Technicians 6. Unemployed or retired 7. Others |
| A8 | The household income levels per year (RMB) | 1. 0-10,000 RMB/Year (0-1,415 USD/Year) 2. 10,000-30,000 RMB/Year (1,415-4,354 USD/Year) 3. 30,000-80,000 RMB/Year (4,354-11,611USD/Year) 4. 80,000-150,000 RMB/Year (11,611-21,771USD/Year) 5. 150,000-300,000 RMB/Year (21771-43541USD/Year) 6. 300,000-1,000,000 RMB/Year (43,541-145,137USD/Year) 7. 1,000,000 RMB/Year or above (145,137 USD /Year or above) |
| A9 | Whether anyone in the same household has ever been infected with HPV or cervical cancer | 1=Yes  2=No  3=Not clear |
| B1 | Today, the researchers introduced the HPV vaccine and the Pay-it-forward project to you. Are you willing for your child receive the HPV vaccine through this project? | 1=Yes  2=No |
| **PART C VACCINE-RELATED INFORMATION** | | |
| C1 | Have you ever heard about the HPV prior to this program | 1=Yes  2=No |
| C2 | Have you ever heard about HPV vaccines prior to this program | 1=Yes  2=No |
| C3 | Have ever heard the piloting projects to cover the HPV vaccination of 13-14 years old girls in Chengdu at the end of 2021 | 1=Yes  2=No |
| C4 | Were you aware of the following vaccines marketed in this country prior to this program |  |
| C4.1 | Were you aware of the following vaccines marketed in this country prior to this program - Nona-valent | 1=Yes  2=No |
| C4.2 | Were you aware of the following vaccines marketed in this country prior to this program – Quadri-valent | 1=Yes  2=No |
| C4.3 | Were you aware of the following vaccines marketed in this country prior to this program - Bi-valent (include both imported and domestic vaccines) | 1=Yes  2=No |
| C5 | In general, I think HPV vaccination is important |  |
| C5.1 | In general, I think nonavalent HPV vaccination is important. | 1. Strongly disagree 2. Disagree 3. Agree 4. Strongly agree |
| C5.2 | In general, I think quadrivalent HPV vaccination is important. | 1. Strongly disagree 2. Disagree 3. Agree 4. Strongly agree |
| C5.3 | In general, I think bivalent HPV vaccination is important (include both imported and domestic vaccines). | 1. Strongly disagree 2. Disagree 3. Agree 4. Strongly agree |
| C6 | In general, I think the HPV vaccination is safe |  |
| C6.1 | In general, I think the nonavalent HPV vaccination is safe. | 1. Strongly disagree 2. Disagree 3. Agree 4. Strongly agree |
| C6.2 | In general, I think the quadrivalent HPV vaccination is safe. | 1. Strongly disagree 2. Disagree 3. Agree 4. Strongly agree |
| C6.3 | In general, I think the bivalent HPV vaccination is safe (include both imported and domestic vaccines). | 1. Strongly disagree 2. Disagree 3. Agree 4. Strongly agree |
| C7 | In general, I think the HPV vaccination is effective |  |
| C7.1 | In general, I think the nonavalent HPV vaccination is effective. | 1. Strongly disagree 2. Disagree 3. Agree 4. Strongly agree |
| C7.2 | In general, I think the quadrivalent HPV vaccination is effective. | 1. Strongly disagree 2. Disagree 3. Agree 4. Strongly agree |
| C7.3 | In general, I think the bivalent HPV vaccination is effective (include both imported and domestic vaccines). | 1. Strongly disagree 2. Disagree 3. Agree 4. Strongly agree |
| C8 | Has anyone around you been vaccinated against HPV? | 1=Yes  2=No |
| C9 | Have you ever been hesitant about getting the HPV vaccination (except for allergies)? | 1=Yes  2=No |
| C10 | Have you ever postponed the HPV vaccination (except for allergies)? | 1=Yes  2=No |
| C11 | Have you ever refused to receive the HPV vaccination (except for allergies)? | 1=Yes  2=No |
| C12 | Have you ever heard about negative information about HPV vaccination | 1=Yes  2=No |
| C13 | Do any of your friends or relatives object to HPV vaccination? | 1=Yes  2=No |
| C14 | Have you or people around you had experienced adverse effects associated with HPV vaccination? | 1=Yes  2=No |
| C15 | Is the cost a barrier for you or your family members to receive the HPV vaccination? | 1=Yes  2=No |
| C16 | Do you think that, after HPV vaccination, your child still needs regular cervical cancer screening after they get married/become sexually active? | 1=Yes  2=No |
| C17 | Which of the above three HPV vaccines would you preferably want your daughter to receive? | 1. 2vHPV vaccines (both imported and domestic) (End) 2. imported 4vHPV vaccines 3. imported 9vHPV vaccines 4. No preferences, all the above are fine (End) 5. Not sure (End) |
| C18 | If your community health center has no supply of 4v/9v HPV vaccines, you will let your daughter | 1=Vaccinate with 2vHPV vaccines (imported or domestic) (End)  2=Not vaccinate now but wait until 4v/9vHPV vaccines are available in the community  3= Others  -3=skip |
| C19 | How long will you be willing to wait in order for your daughter to receive a preferred type of HPV vaccine? | 1=More than five years  2=Two to five years (including five years)  3=One to two years (including two years)  4=Six months to one year (including one year)  5=Six months or below  -3=skip |

# Appendix D. Data analysis code(R·version·4.2.1)

library(margins)

library(readxl)

library(dplyr)

library(janitor)

library(broom)

library(sandwich)

library(xtable)

data <- read_excel("HPV_PIF.xlsx")

data <- clean_names(data)

with(data, table(arm, uptake))

data$arm <- as.numeric(data$arm)

data$uptake <- as.numeric(data$uptake)

data$uptake2 <- as.numeric(data$uptake2)

##Primary outcome

# Adjusted

# education a6

with(data, table(arm, a6))

data$a6<- as.numeric(data$a6)

data <- data %>%

mutate(edu=ifelse(a6>=4, 1, 0))

with(data, table(arm, edu))

# income a8

with(data, table(arm, a8))

data <- data %>%

mutate(income = case_when(

a8==1|a8==2|a8==3 ~ 0,

TRUE ~ 1))

with(data, table(arm,income))

# employment status a7

with(data, table(arm, a7))

data <- data %>%

mutate(unemploy = case_when(

a7==6 ~ 1,

TRUE ~ 0))

with(data, table(arm, unemploy))

# sex of guardian a1

with(data, table(arm, a1))

data <- data %>%

mutate(female = case_when(

a1==2 ~ 1,

TRUE ~ 0))

with(data, table(arm, female))

# study sites

with(data, table(arm, study_site))

# marital status a4

with(data, table(arm, a4))

data <- data %>%

mutate(marital = case_when(

a4==2 ~ 1,

a4==1 ~ 0,

TRUE ~ 2))

with(data, table(arm, marital))

# unadjusted regression

main_unadjusted <- glm(uptake~arm, family = gaussian("identity"), data=data)

tidy(main_unadjusted)[2,]

sqrt(sandwich(main_unadjusted)[2,2])

c(tidy(main_unadjusted)[2,2] - 1.96*sqrt(sandwich(main_unadjusted)[2,2]), tidy(main_unadjusted)[2,2] + 1.96*sqrt(sandwich(main_unadjusted)[2,2]))

# adjusted regression

main_adjusted <- glm(uptake~arm + as.factor(edu) + as.factor(income)

+ as.factor(study_site)+ as.factor(marital) + as.factor(female), family = gaussian("identity"), data=data)

tidy(main_adjusted)[2,]

sqrt(sandwich(main_adjusted)[2,2])

c(tidy(main_adjusted)[2,2] - 1.96*sqrt(sandwich(main_adjusted)[2,2]), tidy(main_adjusted)[2,2] + 1.96*sqrt(sandwich(main_adjusted)[2,2]))

###second dose uptake

# unadjusted regression

main_unadjusted2 <- glm(uptake2~arm, family = gaussian("identity"), data=data)

tidy(main_unadjusted2)[2,]

sqrt(sandwich(main_unadjusted2)[2,2])

c(tidy(main_unadjusted2)[2,2] - 1.96*sqrt(sandwich(main_unadjusted2)[2,2]), tidy(main_unadjusted2)[2,2] + 1.96*sqrt(sandwich(main_unadjusted2)[2,2]))

# adjusted regression

main_adjusted2 <- glm(uptake2~arm + as.factor(edu) + as.factor(income)

+ as.factor(study_site)+ as.factor(marital) + as.factor(female), family = gaussian("identity"), data=data)

tidy(main_adjusted2)[2,]

sqrt(sandwich(main_adjusted2)[2,2])

c(tidy(main_adjusted2)[2,2] - 1.96*sqrt(sandwich(main_adjusted2)[2,2]), tidy(main_adjusted2)[2,2] + 1.96*sqrt(sandwich(main_adjusted2)[2,2]))

## subgroup analyses

# sites

#interaction

model.site <- glm(uptake~arm * study_site + as.factor(edu) + as.factor(income) + as.factor(marital)+ as.factor(female),

family=gaussian(link="identity"), data=data)

summary(model.site)

#site A =yulin

dat1 <- data %>%

filter(study_site==1)

with(dat1, table(arm, uptake))

yulin_unadj <- glm(uptake~arm, family=gaussian(link="identity"), data=dat1)

tidy(yulin_unadj)[2,]

sqrt(sandwich(yulin_unadj)[2,2])

c(tidy(yulin_unadj)[2,2] - 1.96*sqrt(sandwich(yulin_unadj)[2,2]), tidy(yulin_unadj)[2,2] + 1.96*sqrt(sandwich(yulin_unadj)[2,2]))

yulin_adj <- glm(uptake~arm + as.factor(edu) + as.factor(income) + as.factor(marital)+ as.factor(female), family=gaussian(link="identity"), data=dat1)

tidy(yulin_adj)[2,]

sqrt(sandwich(yulin_adj)[2,2])

c(tidy(yulin_adj)[2,2] - 1.96*sqrt(sandwich(yulin_adj)[2,2]), tidy(yulin_adj)[2,2] + 1.96*sqrt(sandwich(yulin_adj)[2,2]))

#site B =longtan

dat2 <- data %>%

filter(study_site==2)

with(dat2, table(arm, uptake))

longtan_unadj <- glm(uptake~arm, family=gaussian(link="identity"), data=dat2)

tidy(longtan_unadj)[2,]

sqrt(sandwich(longtan_unadj)[2,2])

c(tidy(longtan_unadj)[2,2] - 1.96*sqrt(sandwich(longtan_unadj)[2,2]), tidy(longtan_unadj)[2,2] + 1.96*sqrt(sandwich(longtan_unadj)[2,2]))

longtan_adj <- glm(uptake~arm + as.factor(edu) + as.factor(income) + as.factor(marital)+ as.factor(female), family=gaussian(link="identity"), data=dat2)

tidy(longtan_adj)[2,]

sqrt(sandwich(longtan_adj)[2,2])

c(tidy(longtan_adj)[2,2] - 1.96*sqrt(sandwich(longtan_adj)[2,2]), tidy(longtan_adj)[2,2] + 1.96*sqrt(sandwich(longtan_adj)[2,2]))

#site D = xinjin

dat3 <- data %>%

filter(study_site==3)

with(dat3, table(arm, uptake))

xinjin_unadj <- glm(uptake~arm, family=gaussian(link="identity"), data=dat3)

tidy(xinjin_unadj)[2,]

sqrt(sandwich(xinjin_unadj)[2,2])

c(tidy(xinjin_unadj)[2,2] - 1.96*sqrt(sandwich(xinjin_unadj)[2,2]), tidy(xinjin_unadj)[2,2] + 1.96*sqrt(sandwich(xinjin_unadj)[2,2]))

xinjin_adj <- glm(uptake~arm + as.factor(edu) + as.factor(income) + as.factor(marital)+ as.factor(female), family=gaussian(link="identity"), data=dat3)

tidy(xinjin_adj)[2,]

sqrt(sandwich(xinjin_adj)[2,2])

c(tidy(xinjin_adj)[2,2] - 1.96*sqrt(sandwich(xinjin_adj)[2,2]), tidy(xinjin_adj)[2,2] + 1.96*sqrt(sandwich(xinjin_adj)[2,2]))

#site C = dongbu

dat4 <- data %>%

filter(study_site==4)

with(dat4, table(arm, uptake))

dongbu_unadj <- glm(uptake~arm, family=gaussian(link="identity"), data=dat4)

tidy(dongbu_unadj)[2,]

sqrt(sandwich(dongbu_unadj)[2,2])

c(tidy(dongbu_unadj)[2,2] - 1.96*sqrt(sandwich(dongbu_unadj)[2,2]), tidy(dongbu_unadj)[2,2] + 1.96*sqrt(sandwich(dongbu_unadj)[2,2]))

dongbu_adj <- glm(uptake~arm + as.factor(edu) + as.factor(income) + as.factor(marital)+ as.factor(female), family=gaussian(link="identity"), data=dat4)

tidy(dongbu_adj)[2,]

sqrt(sandwich(dongbu_adj)[2,2])

c(tidy(dongbu_adj)[2,2] - 1.96*sqrt(sandwich(dongbu_adj)[2,2]), tidy(dongbu_adj)[2,2] + 1.96*sqrt(sandwich(dongbu_adj)[2,2]))

## sex of guardian

#interaction

model.sex <- glm(uptake~arm * female + as.factor(edu) + as.factor(income) + as.factor(marital) + as.factor(study_site), family=gaussian(link="identity"), data= data

)

summary(model.sex)

model.sex1 <- glm(uptake~arm + as.factor(edu) + as.factor(income) + as.factor(marital) + as.factor(study_site) + arm * female,

family=gaussian(link="identity"), data= data

)

summary(model.sex1)

# female

datf <- data %>%

filter(female==1)

with(datf, table(arm, uptake))

female_unadj <- glm(uptake~arm, family=gaussian(link="identity"), data=datf)

tidy(female_unadj)[2,]

sqrt(sandwich(female_unadj)[2,2])

c(tidy(female_unadj)[2,2] - 1.96*sqrt(sandwich(female_unadj)[2,2]), tidy(female_unadj)[2,2] + 1.96*sqrt(sandwich(female_unadj)[2,2]))

female_adj <- glm(uptake~arm + as.factor(edu) + as.factor(income) + as.factor(marital) + as.factor(study_site), family=gaussian(link="identity"), data=datf)

tidy(female_adj)[2,]

sqrt(sandwich(female_adj)[2,2])

c(tidy(female_adj)[2,2] - 1.96*sqrt(sandwich(female_adj)[2,2]), tidy(female_adj)[2,2] + 1.96*sqrt(sandwich(female_adj)[2,2]))

# male

datm <- data %>%

filter(female==0)

with(datm, table(arm, uptake))

male_unadj <- glm(uptake~arm, family=gaussian(link="identity"), data=datm)

tidy(male_unadj)[2,]

sqrt(sandwich(male_unadj)[2,2])

c(tidy(male_unadj)[2,2] - 1.96*sqrt(sandwich(male_unadj)[2,2]), tidy(male_unadj)[2,2] + 1.96*sqrt(sandwich(male_unadj)[2,2]))

male_adj <- glm(uptake~arm + as.factor(edu) + as.factor(income) + as.factor(marital) + as.factor(study_site), family=gaussian(link="identity"), data=datm)

tidy(male_adj)[2,]

sqrt(sandwich(male_adj)[2,2])

c(tidy(male_adj)[2,2] - 1.96*sqrt(sandwich(male_adj)[2,2]), tidy(male_adj)[2,2] + 1.96*sqrt(sandwich(male_adj)[2,2]))

## age 40#

dataage <- data %>%

mutate(age = ifelse(age<100, age, NA)) %>%

mutate(ageb = case_when(

age<40 & age>0 ~ 1,

age>=40& age<100 ~ 0,

TRUE ~ NA_real_

)

)

with(data, table(arm, a6))

data$a6<- as.numeric(data$a6)

data <- data %>%

mutate(age = ifelse(age<100, age, NA)) %>%

mutate(ageb = case_when(

age<40 & age>0 ~ 1,

age>=40& age<100 ~ 0,

TRUE ~ NA_real_

)

)

with(data, table(arm,ageb))

#interaction

model.age <- glm(uptake~arm * ageb + as.factor(edu) + as.factor(income) + as.factor(marital)+ as.factor(female) + as.factor(study_site),

family=gaussian(link="identity"), data=data)

summary(model.age)

# age <40

age1 <- dataage %>%

filter(ageb==1)

with(age1, table(arm, uptake))

age1_unadj <- glm(uptake~arm, family=gaussian(link="identity"), data=age1)

tidy(age1_unadj)[2,]

sqrt(sandwich(age1_unadj)[2,2])

c(tidy(age1_unadj)[2,2] - 1.96*sqrt(sandwich(age1_unadj)[2,2]), tidy(age1_unadj)[2,2] + 1.96*sqrt(sandwich(age1_unadj)[2,2]))

age1_adj <- glm(uptake~arm + as.factor(edu) + as.factor(income) + as.factor(marital) + as.factor(study_site)+ as.factor(female), family=gaussian(link="identity"), data=age1)

tidy(age1_adj)[2,]

sqrt(sandwich(age1_adj)[2,2])

c(tidy(age1_adj)[2,2] - 1.96*sqrt(sandwich(age1_adj)[2,2]), tidy(age1_adj)[2,2] + 1.96*sqrt(sandwich(age1_adj)[2,2]))

# age >=40

age0 <- dataage %>%

filter(ageb==0)

with(age0, table(arm, uptake))

age0_unadj <- glm(uptake~arm, family=gaussian(link="identity"), data=age0)

tidy(age0_unadj)[2,]

sqrt(sandwich(age0_unadj)[2,2])

c(tidy(age0_unadj)[2,2] - 1.96*sqrt(sandwich(age0_unadj)[2,2]), tidy(age0_unadj)[2,2] + 1.96*sqrt(sandwich(age0_unadj)[2,2]))

age0_adj <- glm(uptake~arm + as.factor(edu) + as.factor(income) + as.factor(marital) + as.factor(study_site)+ as.factor(female), family=gaussian(link="identity"), data=age0)

tidy(age0_adj)[2,]

sqrt(sandwich(age0_adj)[2,2])

c(tidy(age0_adj)[2,2] - 1.96*sqrt(sandwich(age0_adj)[2,2]), tidy(age0_adj)[2,2] + 1.96*sqrt(sandwich(age0_adj)[2,2]))

## income

#interaction

model.income <- glm(uptake~arm * income + as.factor(edu) + as.factor(marital) + as.factor(study_site)+ as.factor(female),

family=gaussian(link="identity"), data=data)

summary(model.income)

# low income

dincome0 <- data %>%

filter(income==0)

with(dincome0, table(arm, uptake))

income0_unadj <- glm(uptake~arm, family=gaussian(link="identity"), data=dincome0)

tidy(income0_unadj)[2,]

sqrt(sandwich(income0_unadj)[2,2])

c(tidy(income0_unadj)[2,2] - 1.96*sqrt(sandwich(income0_unadj)[2,2]), tidy(income0_unadj)[2,2] + 1.96*sqrt(sandwich(income0_unadj)[2,2]))

income0_adj <- glm(uptake~arm + as.factor(edu) + as.factor(marital) + as.factor(study_site)+ as.factor(female), family=gaussian(link="identity"), data=dincome0)

tidy(income0_adj)[2,]

sqrt(sandwich(income0_adj)[2,2])

c(tidy(income0_adj)[2,2] - 1.96*sqrt(sandwich(income0_adj)[2,2]), tidy(income0_adj)[2,2] + 1.96*sqrt(sandwich(income0_adj)[2,2]))

# high income

dincome1 <- data %>%

filter(income==1)

with(dincome1, table(arm, uptake))

income1_unadj <- glm(uptake~arm, family=gaussian(link="identity"), data=dincome1)

tidy(income1_unadj)[2,]

sqrt(sandwich(income1_unadj)[2,2])

c(tidy(income1_unadj)[2,2] - 1.96*sqrt(sandwich(income1_unadj)[2,2]), tidy(income1_unadj)[2,2] + 1.96*sqrt(sandwich(income1_unadj)[2,2]))

income1_adj <- glm(uptake~arm + as.factor(edu) + as.factor(marital) + as.factor(study_site)+ as.factor(female), family=gaussian(link="identity"), data=dincome1)

tidy(income1_adj)[2,]

sqrt(sandwich(income1_adj)[2,2])

c(tidy(income1_adj)[2,2] - 1.96*sqrt(sandwich(income1_adj)[2,2]), tidy(income1_adj)[2,2] + 1.96*sqrt(sandwich(income1_adj)[2,2]))

## education

#interaction

model.edu <- glm(uptake~arm * edu + as.factor(income) + as.factor(marital) + as.factor(study_site)+ as.factor(female),

family=gaussian(link="identity"), data=data)

summary(model.edu)

# college or higher

date1 <- data %>%

filter(edu==1)

with(date1, table(arm, uptake))

edu1_unadj <- glm(uptake~arm, family=gaussian(link="identity"), data=date1)

tidy(edu1_unadj)[2,]

sqrt(sandwich(edu1_unadj)[2,2])

c(tidy(edu1_unadj)[2,2] - 1.96*sqrt(sandwich(edu1_unadj)[2,2]), tidy(edu1_unadj)[2,2] + 1.96*sqrt(sandwich(edu1_unadj)[2,2]))

edu1_adj <- glm(uptake~arm + as.factor(income) + as.factor(marital) + as.factor(study_site)+ as.factor(female), family=gaussian(link="identity"), data=date1)

tidy(edu1_adj)[2,]

sqrt(sandwich(edu1_adj)[2,2])

c(tidy(edu1_adj)[2,2] - 1.96*sqrt(sandwich(edu1_adj)[2,2]), tidy(edu1_adj)[2,2] + 1.96*sqrt(sandwich(edu1_adj)[2,2]))

# high school or below

date0 <- data %>%

filter(edu==0)

with(date0, table(arm, uptake))

edu0_unadj <- glm(uptake~arm, family=gaussian(link="identity"), data=date0)

tidy(edu0_unadj)[2,]

sqrt(sandwich(edu0_unadj)[2,2])

c(tidy(edu0_unadj)[2,2] - 1.96*sqrt(sandwich(edu0_unadj)[2,2]), tidy(edu0_unadj)[2,2] + 1.96*sqrt(sandwich(edu0_unadj)[2,2]))

edu0_adj <- glm(uptake~arm + as.factor(income) + as.factor(marital) + as.factor(study_site)+ as.factor(female), family=gaussian(link="identity"), data=date0)

tidy(edu0_adj)[2,]

sqrt(sandwich(edu0_adj)[2,2])

c(tidy(edu0_adj)[2,2] - 1.96*sqrt(sandwich(edu0_adj)[2,2]), tidy(edu0_adj)[2,2] + 1.96*sqrt(sandwich(edu0_adj)[2,2]))

###awareness of HPV vaccines

with(data, table(arm, c2))

data$c2<- as.numeric(data$c2)

data <- data %>%

mutate(hpv=ifelse(c2==1, 1, 0))

with(data, table(arm, hpv))

#interaction

model.hpv <- glm(uptake~arm * hpv + as.factor(income) + as.factor(marital) + as.factor(study_site)+ as.factor(female),

family=gaussian(link="identity"), data=data)

summary(model.hpv)

## know the HPV vaccine

dathpv1 <- data %>%

filter(hpv==1)

with(dathpv1, table(arm, uptake))

hpv1_unadj <- glm(uptake~arm, family=gaussian(link="identity"), data=dathpv1)

tidy(hpv1_unadj)[2,]

sqrt(sandwich(hpv1_unadj)[2,2])

c(tidy(hpv1_unadj)[2,2] - 1.96*sqrt(sandwich(hpv1_unadj)[2,2]), tidy(hpv1_unadj)[2,2] + 1.96*sqrt(sandwich(hpv1_unadj)[2,2]))

hpv1_adj <- glm(uptake~arm +as.factor(edu) + as.factor(income)

+ as.factor(study_site)+ as.factor(marital) + as.factor(female), family=gaussian(link="identity"), data=dathpv1)

tidy(hpv1_adj)[2,]

sqrt(sandwich(hpv1_adj)[2,2])

c(tidy(hpv1_adj)[2,2] - 1.96*sqrt(sandwich(hpv1_adj)[2,2]), tidy(hpv1_adj)[2,2] + 1.96*sqrt(sandwich(hpv1_adj)[2,2]))

## not know the HPV vaccine

dathpv2 <- data %>%

filter(hpv==0)

with(dathpv2, table(arm, uptake))

hpv2_unadj <- glm(uptake~arm, family=gaussian(link="identity"), data=dathpv2)

tidy(hpv2_unadj)[2,]

sqrt(sandwich(hpv2_unadj)[2,2])

c(tidy(hpv2_unadj)[2,2] - 1.96*sqrt(sandwich(hpv2_unadj)[2,2]), tidy(hpv2_unadj)[2,2] + 1.96*sqrt(sandwich(hpv2_unadj)[2,2]))

hpv2_adj <- glm(uptake~arm + as.factor(income) + as.factor(marital) + as.factor(study_site)+ as.factor(female)+ as.factor(edu), family=gaussian(link="identity"), data=dathpv2)

tidy(hpv2_adj)[2,]

sqrt(sandwich(hpv2_adj)[2,2])

c(tidy(hpv2_adj)[2,2] - 1.96*sqrt(sandwich(hpv2_adj)[2,2]), tidy(hpv2_adj)[2,2] + 1.96*sqrt(sandwich(hpv2_adj)[2,2]))

####secondary outcome

# secondary outcome: vaccine delay

data_vcc <- data %>%

filter(b1 == 1) %>%

mutate(vcc = case_when(

(b1 == 1 & uptake == 0) ~ 1,

TRUE ~ 0

))

with(data_vcc, table(arm, vcc))

# crude

vcc_unadj <- glm(vcc~arm, family=gaussian(link="identity"), data=data_vcc)

tidy(vcc_unadj)[2,]

sqrt(sandwich(vcc_unadj)[2,2])

c(tidy(vcc_unadj)[2,2] - 1.96*sqrt(sandwich(vcc_unadj)[2,2]), tidy(vcc_unadj)[2,2] + 1.96*sqrt(sandwich(vcc_unadj)[2,2]))

# adjusted

vcc_adj<- glm(vcc~arm + + as.factor(income) + as.factor(marital) +

as.factor(study_site)+ as.factor(female)+ as.factor(edu), family=gaussian(link="identity"), data=data_vcc)

tidy(vcc_adj)[2,]

sqrt(sandwich(vcc_adj)[2,2])

c(tidy(vcc_adj)[2,2] - 1.96*sqrt(sandwich(vcc_adj)[2,2]), tidy(vcc_adj)[2,2] + 1.96*sqrt(sandwich(vcc_adj)[2,2]))

# vaccine confidence

data_vc <- data %>%

mutate_at(

vars(one_of('c5_1', 'c5_3','c6_1', 'c6_3','c7_1', 'c7_3')),

funs(case_when(

. == 3 | . == 4 ~ 1,

. == 1 | . == 2 ~ 0)))

# importancy v9

with(data_vc, table(arm, c5_1))

# crude

v9impor_unadj <- glm(c5_1~arm, family=gaussian(link="identity"), data=data_vc)

tidy(v9impor_unadj)[2,]

sqrt(sandwich(v9impor_unadj)[2,2])

c(tidy(v9impor_unadj)[2,2] - 1.96*sqrt(sandwich(v9impor_unadj)[2,2]), tidy(v9impor_unadj)[2,2] + 1.96*sqrt(sandwich(v9impor_unadj)[2,2]))

# adjusted regression

v9impor_adj <- glm(c5_1~arm + as.factor(income) + as.factor(marital) +

as.factor(study_site)+ as.factor(female)+ as.factor(edu), family=gaussian(link="identity"), data=data_vc)

tidy(v9impor_adj)[2,]

sqrt(sandwich(v9impor_adj)[2,2])

c(tidy(v9impor_adj)[2,2] - 1.96*sqrt(sandwich(v9impor_adj)[2,2]), tidy(v9impor_adj)[2,2] + 1.96*sqrt(sandwich(v9impor_adj)[2,2]))

# importancy v2

with(data_vc, table(arm, c5_3))

# crude

v2impor_unadj <- glm(c5_3~arm, family=gaussian(link="identity"), data=data_vc)

tidy(v2impor_unadj)[2,]

sqrt(sandwich(v2impor_unadj)[2,2])

c(tidy(v2impor_unadj)[2,2] - 1.96*sqrt(sandwich(v2impor_unadj)[2,2]), tidy(v2impor_unadj)[2,2] + 1.96*sqrt(sandwich(v2impor_unadj)[2,2]))

# adjusted regression

v2impor_adj <- glm(c5_3~arm + as.factor(income) + as.factor(marital) +

as.factor(study_site)+ as.factor(female)+ as.factor(edu), family=gaussian(link="identity"), data=data_vc)

tidy(v2impor_adj)[2,]

sqrt(sandwich(v2impor_adj)[2,2])

c(tidy(v2impor_adj)[2,2] - 1.96*sqrt(sandwich(v2impor_adj)[2,2]), tidy(v2impor_adj)[2,2] + 1.96*sqrt(sandwich(v2impor_adj)[2,2]))

# safety v9

with(data_vc, table(arm, c6_1))

# crude

v9safe_unadj <- glm(c6_1~arm, family=gaussian(link="identity"), data=data_vc)

tidy(v9safe_unadj)[2,]

sqrt(sandwich(v9safe_unadj)[2,2])

c(tidy(v9safe_unadj)[2,2] - 1.96*sqrt(sandwich(v9safe_unadj)[2,2]), tidy(v9safe_unadj)[2,2] + 1.96*sqrt(sandwich(v9safe_unadj)[2,2]))

# adjusted regression

v9safe_adj <- glm(c6_1~arm + as.factor(income) + as.factor(marital) +

as.factor(study_site)+ as.factor(female)+ as.factor(edu), family=gaussian(link="identity"), data=data_vc)

tidy(v9safe_adj)[2,]

sqrt(sandwich(v9safe_adj)[2,2])

c(tidy(v9safe_adj)[2,2] - 1.96*sqrt(sandwich(v9safe_adj)[2,2]), tidy(v9safe_adj)[2,2] + 1.96*sqrt(sandwich(v9safe_adj)[2,2]))

# safety v2

with(data_vc, table(arm, c6_3))

# crude

v2safe_unadj <- glm(c6_3~arm, family=gaussian(link="identity"), data=data_vc)

tidy(v2safe_unadj)[2,]

sqrt(sandwich(v2safe_unadj)[2,2])

c(tidy(v2safe_unadj)[2,2] - 1.96*sqrt(sandwich(v2safe_unadj)[2,2]), tidy(v2safe_unadj)[2,2] + 1.96*sqrt(sandwich(v2safe_unadj)[2,2]))

# adjusted regression

v2safe_adj <- glm(c6_3~arm + as.factor(income) + as.factor(marital) +

as.factor(study_site)+ as.factor(female)+ as.factor(edu), family=gaussian(link="identity"), data=data_vc)

tidy(v2safe_adj)[2,]

sqrt(sandwich(v2safe_adj)[2,2])

c(tidy(v2safe_adj)[2,2] - 1.96*sqrt(sandwich(v2safe_adj)[2,2]), tidy(v2safe_adj)[2,2] + 1.96*sqrt(sandwich(v2safe_adj)[2,2]))

# effectiveness v9

with(data_vc, table(arm, c7_1))

# crude

v9effect_unadj <- glm(c7_1~arm, family=gaussian(link="identity"), data=data_vc)

tidy(v9effect_unadj)[2,]

sqrt(sandwich(v9effect_unadj)[2,2])

c(tidy(v9effect_unadj)[2,2] - 1.96*sqrt(sandwich(v9effect_unadj)[2,2]), tidy(v9effect_unadj)[2,2] + 1.96*sqrt(sandwich(v9effect_unadj)[2,2]))

# adjusted regression

v9effect_adj <- glm(c7_1~arm + as.factor(income) + as.factor(marital) +

as.factor(study_site)+ as.factor(female)+ as.factor(edu), family=gaussian(link="identity"), data=data_vc)

tidy(v9effect_adj)[2,]

sqrt(sandwich(v9effect_adj)[2,2])

c(tidy(v9effect_adj)[2,2] - 1.96*sqrt(sandwich(v9effect_adj)[2,2]), tidy(v9effect_adj)[2,2] + 1.96*sqrt(sandwich(v9effect_adj)[2,2]))

# effectiveness v2

with(data_vc, table(arm, c7_3))

# crude

v2effect_unadj <- glm(c7_3~arm, family=gaussian(link="identity"), data=data_vc)

tidy(v2effect_unadj)[2,]

sqrt(sandwich(v2effect_unadj)[2,2])

c(tidy(v2effect_unadj)[2,2] - 1.96*sqrt(sandwich(v2effect_unadj)[2,2]), tidy(v2effect_unadj)[2,2] + 1.96*sqrt(sandwich(v2effect_unadj)[2,2]))

# adjusted regression

v2effect_adj <- glm(c7_3~arm + as.factor(income) + as.factor(marital) +

as.factor(study_site)+ as.factor(female)+ as.factor(edu), family=gaussian(link="identity"), data=data_vc)

tidy(v2effect_adj)[2,]

sqrt(sandwich(v2effect_adj)[2,2])

c(tidy(v2effect_adj)[2,2] - 1.96*sqrt(sandwich(v2effect_adj)[2,2]), tidy(v2effect_adj)[2,2] + 1.96*sqrt(sandwich(v2effect_adj)[2,2]))

# Appendix E. Video abstract

<https://drive.google.com/file/d/1eiiuu1ihXjuBZremMWBqs06A5liuqHEX/view?usp=drive_link>

**Cost analysis section**

We compared costs between all two arms and present the collected costs data (Table B in S1 File) and analyzed costs (Figure F in S1 File). Financial costs per person are reported in the main text.

# Table A. Cost calculations (in 2022 USD) for the study

| **Group** | **PIF** | **SOC** |
| --- | --- | --- |
| **Item** | Cost | Cost |
| **Start-up cost** |  |  |
| ***Training of clinic staff, travel to training site*** |  |  |
| Trainers （Experts) | 200.2 | 200.2 |
| Trainers （MSc student.) | 7.3 | 7.3 |
| ***Community engaged postcards design*** |  |  |
| Participants | 375.8 | \ |
| ***Consultation workshop*** |  |  |
| Expert consultation | 428.6 | 277.3 |
| Subtotal | 1011.8 | 484.8 |
|  |  |  |
| **Fixed cost** |  |  |
| ***Recruitment-independent personnel cost*** |  |  |
| Vaccinators | 897.2 | 456.7 |
| Administration staff | 893.0 | 454.6 |
| Clinic coordinator | 1687.5 | 1677.0 |
| Community workers | 1661.8 | 1651.5 |
| ***Work and management expenses*** |  |  |
| Internet rent fee | 3.3 | 3.3 |
| Power expense | 15.3 | 15.3 |
| Telephone airtime charge | 75.2 | 75.2 |
| Text fee | 23.9 | 23.9 |
| WenJuanXing platform rent for data collection | 28.9 | 28.9 |
| ***Travel expenses*** |  |  |
| Accommodation | 1099.8 | 1099.8 |
| Transport costs | 118.1 | 118.1 |
| ***Supply*** |  |  |
| Envelope | 24.7 | 24.7 |
| Pen | 1.5 | 1.5 |
| Subtotal | 6530.1 | 5630.5 |
|  |  |  |
| **Variable Cost** |  |  |
| Research assistant | 2901.7 | 2176.3 |
| Informed consent printing | 6.4 | 6.4 |
| 2vHPV vaccine | 2625.3 | 1336.5 |
| Postcard | 58.0 | \ |
| Intro pamphlets, roll-up banner | 23.4 | 23.2 |
| Donation box | 11.2 | \ |
| Subtotal | 5626.1 | 3542.4 |
|  |  |  |
| **Contributions*** | 491.7 | 1417.8 |
| *In the PIF arm, "contribution" refers to the sum of donations and service fees, while in the SOC group, it denotes self-payment by participants. | | |

# Table B. Recruitment, appointment, and reasons for non-presence for vaccination

|  | Number of recruits | | Number of scheduled vaccinations  (n, %) | | Number of non-presence for vaccination  (n, %) | | Reasons for non-presence for vaccination | | | | |
| --- | --- | --- | --- | --- | --- | --- | --- | --- | --- | --- | --- |
|  | SOC | PIF | SOC | PIF | SOC | PIF | SOC | | PIF | |  |
|  |  |  |  |  |  |  | Refused bivalent HPV vaccine (n, %) | Lost-to-follow-up (n, %) | Refused bivalent HPV vaccine (n, %) | Lost-to-follow-up (n, %) |  |
| Site A | 40 | 43 | 3 | 17 | 1 | 6 | 1 | 0 | 4 | 2 |  |
| Site B | 44 | 39 | 11 | 19 | 0 | 1 | 0 | 0 | 0 | 1 |  |
| Site C | 38 | 39 | 12 | 19 | 4 | 2 | 0 | 4 | 0 | 2 |  |
| Site D | 38 | 40 | 9 | 13 | 2 | 4 | 2 | 0 | 1 | 3 |  |
| Total | 160 | 161 | 35 | 68 | 7 | 13 | 3 | 4 | 5 | 8 |  |

**REFERENCES**

1. Falcaro M, Castañon A, Ndlela B, Checchi M, Soldan K, Lopez-Bernal J, et al. The effects of the national HPV vaccination programme in England, UK, on cervical cancer and grade 3 cervical intraepithelial neoplasia incidence: a register-based observational study. Lancet. 2021;398(10316):2084–92. Available from: https://doi.org/10.1016/S0140-6736(21)02178-4

2. Yang J, Shen M, Wang Z, et al. Prevalence and influencing factors of sexual behavior among university freshmen. Chinese Journal Public Health 2021; 37(03): 431-4.
