# Supplementary material for: Effect of a pay-it-forward strategy on reducing HPV vaccine delay and increasing uptake among 15- to 18-year-old girls in China: A randomized controlled trial
Source: PLoS Med. 2025 Jul 31;22(7):e1004535. doi: 10.1371/journal.pmed.1004535 (PMC12331080; doi:10.1371/journal.pmed.1004535)
Supplement: Attachment — Submitted filename: final_R1_author response_PlosMed_May2025.docx [file pmed.1004535.s008.docx]

# Author response

## Response to academic editor

**Comment**: Please provide a more in-depth discussion on the translation of these results to other contexts.

**Response:**

Thank you for your comment. The pay-it-forward strategy is a community-engaged intervention that leverages prosocial behavior and kindness to promote health behavior uptake. A recent review (ref) has shown that prosocial interventions have been implemented across diverse contexts—including the United States, China, Canada, Japan, Colombia, and Chile—demonstrating their adaptability to different cultural and healthcare settings. While most existing applications of prosocial interventions have focused on mental health outcomes, their use in promoting health behaviors such as vaccination is still emerging and requires further research. Our findings contribute to this growing area and suggest potential for broader application, though local adaptation and stakeholder engagement would be essential for successful translation.

We have added a brief discussion in the lines 401-404, which reads: “*A systematic review demonstrated that pro-social interventions are associated with improved health outcomes for both givers and recipients and have been implemented across diverse settings, highlighting their adaptability to various cultural and healthcare contexts*”.

Ref: Byrne M, Tan RKJ, Wu D, Marley G, Hlatshwako TG, Tao Y, et al., "Prosocial interventions and health outcomes: A systematic review and meta-analysis," JAMA Netw Open 6 (2023):e2346789-e2346789, <http://dx.doi.org/10.1001/jamanetworkopen.2023.46789>.

## Response to reviewer 1

**Comment 1**

P9, L189. Just wondering if the wording about timing 'within three months after receiving the intervention' could be adjusted, as one study arm is usual care, so perhaps should be 'after randomisation' or 'first contact'?

**Response:**

Thank you for the suggestion. We agree with your point and have revised the sentence to read: “*The primary outcome of the study was first-dose HPV vaccine uptake within three months after randomization*”. (lines 222-223)

**Comment 2:**

P11, L214. When was Fisher's exact test used over Chi square?

**Response:**

Thank you for pointing this out. We did not use Fisher's exact test in our analysis. We have revised the sentence to clarify: “*The pay-it-forward and control arms’ uptake rates were compared using chi-square tests*”. (lines 252-253)

**Comment 3:**

P11, L216. A limitation of using the linear probability model (i.e. glm with Gaussian dist and robust error) to estimate risk difference is that the correction that 'sandwich' part of the error provides is sensitive to sample size, with a large sample size needed to avoid a biased SE. Can you confirm that for the main outcome, the CI for crude difference in proportion is comparable to a Newcombe CI for risk difference? (https://doi.org/10.1002/(SICI)1097-0258(19980430)17:8%3C873::AID-SIM779%3E3.0.CO;2-I Method 10/11). Alternatively, there are methods that can estimate risk differences in a multivariable model (e.g. margins command in Stata, and the margins package in R).

**Response:**

Thank you for this insightful comment. In response, we used the *margins* package in R to re-estimate the primary outcome. The recalculated adjusted risk difference was 17.9% (95% CI: 8.4%–27.3%), which is comparable to the estimate obtained using the linear probability model (17.9%, 95% CI: 8.7%–27.0%).

**Comment 4:**

P15. The p-value provided for the sub-group comparisons is inconsistent with the risk difference estimates for each level of the subgroup, e.g. the CIs for sex of caregiver show a high degree of overlap, but the p-value reported is <0.001. It's not currently reported in the methods how the sub-group analyses were completed - these should be analysed by an interaction between treatment variable, and sub-group variable, with the p-value for this parameter the test of heterogeneity in treatment according to the sub-group levels.

**Response:**

Thank you for your thoughtful comment. We agree that subgroup analyses should be interpreted using interaction terms. We have now conducted formal interaction analyses between the intervention and each subgroup variable, and the p-values for these interaction terms are reported in Table 2. To clarify this approach, we have added the following sentence to the Results section: “*Interaction p-values are reported in Table 2 to assess heterogeneity of treatment effects across subgroups.*” (lines 328-330).

Additionally, we have removed p-values for individual subgroup effects to prevent potential misinterpretation and enhance clarity.

**Comment 5:**

Table 1. The footnote has 'Data are mean (SD)' but it looks like SD is presented with a '±' symbol. I'd suggest presenting it in brackets instead.

**Response:**

Thank you for the helpful suggestion. We have revised the table to present standard deviations in parentheses rather than using the “±” symbol in Table 1.

## Response to reviewer 2

**Comment 1:**

Mention that eligible were unvaccinated girls, and what the setting of the study was community health centers)

**Response:**

Thank you for the suggestion. We have added this information to the abstract. It now reads: “*Eligible participants were unvaccinated girls living in the service areas of community health centers (CHCs), and were randomly assigned to either the pay-it-forward arm or the control arm (self-paid vaccination)*.” (lines 47-49)

**Comment 2:**

The costs in the pay-forward strategy are 28% higher: please correct the « at comparable costs », eg, «at one quarter higher costs».

**Response:**

We appreciate your feedback. We have removed any reference to “comparable cost” throughout the manuscript. The sentence now reads: “*The pro-social pay-it-forward strategy was effective to increase catch-up HPV vaccination among teenage girls*”. (lines 59-60)

**Comment 3:**

Please mention how many doses are recommended in China at different age groups.

**Response**

We have added this information in the manuscript, which now states: “*In China, two doses of the HPV vaccine are recommended for girls aged 9–14, while three doses are required for girls in the catch-up age group.*” (lines 104-106)

**Comment4:**

Line 81: «fewer doses» makes sense only if 3 doses were recommended for catch-up

**Response:**

Thank you for pointing this out. We have clarified the recommendation: “*In China, two doses of the HPV vaccine are recommended for girls aged 9–14, while three doses are required for girls in the catch-up age group*.” (lines 104-106)

**Comment5:**

Line 82: What is delayed vaccination a barrier for? please revise the sentence.

**Response:**

We have revised the sentence to clarify the implication of vaccine delay: “*vaccine delay — defined as receiving the vaccination after the recommended age — increases the risk of HPV infection*.” (lines 108-109)

**Comment 6:**

Line 86: Please revise the sentence « the belief that their girls are sexually inactive ». At age 13-14, when parents delay vaccination despite the recommendation, most girls will indeed be sexually inactive. Reproduce the cited results as closely as possible.

**Response:**

We have revised the sentence to better reflect the cited findings: “*but 80% [16] choose to delay vaccination at a later age due to poor awareness, assuming that girls under 18 face minimal risk since they are mostly at home or in school [14].*” (lines 112-114)

**Comment7:**

Line 87: « a community-engaged solution …. ». Please revise the introduction to clarify the justification to work on the catch-up. So far, the state of the art addressed reasons for delay beyond age 14, but not factors related to missing catch-up. Are there any specific data for the reasons, if not please just say that you suppose that the reasons are the same for the initial non-adherence. Then please introduce the idea of community-engagement: what is this and why do you think it may be a solution, given evidence in Chinese population?

**Response:**

We have revised the introduction accordingly. The following sentences have been added: “*Some local governments in China have introduced subsidized HPV vaccination programs for girls aged 13–14, but these exclude catch-up vaccinations for older girls (15–18), leaving low coverage rates (<15%) among this age group [9, 10]. To achieve the cervical cancer elimination goal, catch-up age groups should also be included [11]..*” (lines 99-102)

“*Community engagement involves building partnerships that empower stakeholders to work together in addressing health challenges and promoting overall well-being [16]. Community engaged interventions have been shown to be effective in increasing the vaccine uptake* *[17]*.” (lines 115-118)

**Comment 8:**

Line 89: please provide a specific reference to the nudge terminology.

**Response**

We have added a reference to support the use of nudge theory terminology:

Arno, A., & Thomas, S. (2016). *The efficacy of nudge theory strategies in influencing adult dietary behaviour: a systematic review and meta-analysis*. BMC Public Health, 16, 676. <https://doi.org/10.1186/s12889-016-3272-x>

**Comment 9:**

Please explain what the donations were being used for and in which way. Eg, are they given to specific users (according to which rules?), or integrated in the health center budget? In absence of this information - and given the eventually small donations made (relative to the vaccine price and the subsidy claim) -, it is not clear whether this can really be called a pay-it-forward strategy.

**Response:**

Thank you for the important question. Donations were integrated into the program’s operational budget and used to support future HPV vaccinations for eligible girls in the same community. While individual donations may be small relative to the vaccine cost, they serve as a key component of the prosocial mechanism—highlighting voluntary generosity and fostering a sense of collective support. The primary goal of using the model in this project is to promote vaccine uptake through kindness and altruism(ref).

Ref: Byrne M, Tan RKJ, Wu D, Marley G, Hlatshwako TG, Tao Y, et al., "Prosocial interventions and health outcomes: A systematic review and meta-analysis," JAMA Netw Open 6 (2023):e2346789-e2346789, <http://dx.doi.org/10.1001/jamanetworkopen.2023.46789>.

**Comment 10:**

Another point is that social inequalities are not at all addressed, although financial barriers are presented as essential and part of the lever of the evaluated strategy. Please present a few data from China.

**Response:**

We agree this is an important issue. Based on our data (Table 2), participants from Sites A and B—more economically developed areas—had higher donation rates and contributed larger amounts. This suggests the potential of the pay-it-forward strategy to enable individuals in better-off areas to support those in underserved communities. Although this strategy may not directly reduce systemic social inequalities, it holds promise in promoting solidarity and facilitating access to vaccination through community solidarity.

**Comment 11:**

Which proportion of girls living in the area or a health center usually enrols in the lists used for recruitment, are there any specific characteristics of the population doing so (lower income)?

**Response:**

Among the 1840 contactable eligible girls, about 36.0% (662/1840) were enrolled in this program. Unfortunately, public population-level characteristics data are not available. We are therefore unable to compare between our project participants and the general population in the region. We acknowledged in the discussion section that “*Selection bias caused by convenience sampling of study sites is possible, and generalizability to the general population should be made cautiously. But our study sites from different economic settings reflected common pathways for HPV vaccination in China.*” (lines 422-425)

**Comment 12:**

Line 124: « An individual-based randomization approach … was appropriate. » This statement is not clear. Please clarify what would have been the alternative and justify your judgment. Reference 24 is not complete.

**Response:**

We have clarified the sentence, which now reads: “*An individual-based randomization approach was appropriate for the efficacy evaluation of a newly developed strategy and is more cost-effective*” (lines 177-189).

We also added the complete reference for reference 24: Bhide A, Shah PS, Acharya G. A simplified guide to randomized controlled trials. Acta Obstet Gynecol Scand. 2018;97(4):380-7.

**Comment 13:**

Line 135: How was the purpose of the visit at the center presented to the families? Any notion of HPV, of a randomised study? Where and when did caregivers provide consent, before or during the visit?

**Response:**

The purpose of the study and the visit at the center was explained during the telephone recruitment, and the telephone recruitment script is available in the Appendix A in S1 File. During the telephone recruitment process, participants were given a brief introduction to HPV and cervical cancer, and were informed about the 50% chance of being assigned to the pay-it-forward arm or the control arm.

We revised the sentence in the manuscript: “*Participating caregivers provided verbal consent during telephone recruitment and signed a written informed consent form, and girls provided assent during the on-site visit*.” (lines 166-168).

**Comment 14:**

Line 159: Please clarify: when did families in the intervention arm receive the post-card message? During the consultation at the center, or later via mail? Who gave or sent the postcard?

**Response:**

We have clarified that the participants received postcards during the intervention process and were invited by project staff to write anonymous messages to support others: “*In addition to the routine educational pamphlet, caregiver-girl pairs in the pay-it-forward arm received the intervention that included community co-created postcard messages, featuring messages from previous participants on the back.*” (lines 190-192).

**Comment 15:**

Line 175: when did girls receive the postcards? During the vaccination consultation, or before? Are these the same as those seen by caregivers during the first consultation?

**Response:**

We have clarified that the girls received the postcards during the half-hour post-vaccine monitoring period, and the postcards were the same as those seen by caregivers during the first consultation. The sentence now reads: “*In the pay-it-forward arm, during the half-hour post-vaccine monitoring period, girls received subsidized vaccination and handwritten postcards and they had an opportunity to create their own postcard messages for others, while caregivers were asked about donation.*”. (lines 206-209)

**Comment 16:**

Line 181: please explain what « information sheet » is to track vaccine uptake and adverse events. Wasn't this given to vaccinated girls, only?

**Response:**

We collected vaccine uptake data verified by clinical records, which mainly included: 1) Whether the consented participants received the HPV vaccine; 2) The time of vaccine uptake; 3) Whether any adverse events occurred within 24 hours.

**Comment 17:**

Line 182: when did parents answer the questionnaire about their vaccine attitudes? How was the information on donation behaviour collected? From which « expense records » where the information collected?

**Response:**

Participants completed an online questionnaire including items on vaccine attitudes after they received the project information and intervention materials. The donation information was cross-checked and verified using the official project account hosted on WeChat (a mobile app with monetary transaction function), and handwritten postcard messages were collected through an anonymous donation box. Regarding data for cost analyses, some expenses were documented in project payment records, and some cost data were part of the community's fixed assets, which we collected from local staff. The data collection section now reads: “*We used information tracking sheet to collect data about vaccine uptake and adverse events within 24 hours, which were verified against clinical records. All participating caregivers completed an online questionnaire (Appendix C in S1 File) on demographic information and vaccine attitudes after* *they received the project information and intervention materials. The number of participants who donated, and the donation amount in the pay-it-forward arm were* *verified against the official project account hosted on WeChat (a mobile app with monetary transaction function). The cost data were obtained from project expense records and by asking local health staff*.” (lines 213-220).

**Comment 18:**

Please describe more precisely how vaccine delay was assessed.

**Response:**

We have added a more detailed explanation of the vaccine delay assessment: “*Vaccine delay refers to individuals have positive intentions to vaccinate but fail to act.[29]. The measurement of vaccine delay was adapted from a study evaluating COVID-19 vaccine delay.[30]. We asked participants' willingness to receive the vaccine, and the actual vaccination was verified using clinical records after the 3-month follow-up period. If participants expressed willingness to receive the vaccine but did not obtain it by the end of the follow-up period, this was operationally defined as vaccine delay*.” (lines 231-236)

**Comment 19:**

Line 190: Why do you specify that the vaccine supply was stable ONLY during the period? If supply is an issue, then this should be mentioned in the discussion about external validity of the results, not here.

**Response:**

Sorry about the confusion. We removed “only” from the sentence, and the sentence reads as: “*The four community health centers had a stable supply of bivalent HPV vaccine (**Cecolin®) during the study period*.” (lines 223-224)

**Comment 20:**

Line 192: How was the second dose organised? Please specify the circumstances for this secondary outcome….

**Response:**

We have clarified as: “*Information on second-dose HPV vaccine uptake was collected from clinical records, with no additional intervention administered after the first dose*.” (lines 228-230)

**Comment 21:**

Line 210: Please link the sample size of 160 per arm to the 80 per center that was mentioned previously.

**Response:**

We have added the sentence: “*Based on the structure of our study, with four centers participating, the sample size of 160 per arm corresponds to approximately 80 participants per center (40 in the control arm and 40 in the intervention arm).*” (lines 246-249)

**Comment 22:**

How were vaccine confidence data analysed? Please conduct effectiveness analyses stratified by vaccine confidence levels.

**Response:**

Thank you for your suggestion. We did not collect vaccine confidence data at baseline; therefore, it is not possible to conduct stratified effectiveness analyses by baseline confidence. We recognize this as a limitation. However, individual randomization helps balance potential confounders, including vaccine confidence, between arms. Vaccine confidence was assessed as a secondary outcome during follow-up.

**Comment 23:**

Line 252: What does « successfully invite » refer to, if the response rate is 52% ? Please revise.

**Response:**

We have clarified that “*a total of 662 eligible caregiver/daughter pairs verbally agreed to attend clinics via telephone recruitment, and 360 were present for eligibility screening at clinics*”. (lines 291-293)

**Comment 24:**

Also, which percentage of the no-shows were vaccinated? If all, then the 48% los should be presented as a major barrier of the intervention feasibility. Please discuss whether the intervention can be integrated in other center visits (routine or for specific health issues).

**Response:**

Unfortunately, we were not able to collect information about their vaccination status without their consent to participate in the study. We agree that solutions to improve participants’ willingness to participate in health projects and access vaccination services will be important. Our group are currently conducting research to explore the feasibility of integrating these solutions into routine clinic and school-based vaccination programs via collaborating with local CDCs and community health centers. We hope to share more insights in the near future.

**Comment 25:**

Line 261: Please include information on baseline vaccine confidence in the participant characteristics table. If this was not assessed at baseline, please explain why and discuss the related limitation (any risk for difference observed at follow-up already existing at baseline). Also mention in Figure 3 that these are follow-up data, without adjustment for baseline characteristics.

**Response:**

Thank you for your constructive suggestions. Vaccine confidence was not assessed at baseline. We assume that the individual-level randomization method likely balanced baseline participant characteristics between arms including vaccine confidence levels, but future research could consider measuring pre- and post-intervention vaccine confidence.

All secondary outcome data were collected during follow-up. The analyses were adjusted for participant characteristics including study site, household income level, education level, marital status, and sex of the caregiver. We have added a note to Figure 3 stating: “*All secondary outcome data were collected during follow-up*.”

**Comment 26:**

Line 323: please inform on the costs that increased for the healthcare provider.

**Response:**

As healthcare providers were required to perform additional tasks at intervention sites, related costs increased. Detailed cost information is provided in the Table A in S1 File.

**Comment 27:**

Line 334: rather « encourage to get vaccinated »

**Response:**

We have revised the phrase to: “*encourage to get vaccinated*”. (lines 353)

**Comment 28:**

Line 340: please make clear that the results pertain to catch-up vaccine uptake and provide a short characterisation of the study setting

**Response:**

We have clarified in the background that catch-up vaccine uptake refers to girls aged 15–18 who missed the government-subsidized HPV vaccination offered to 13–14-year-olds. The sentence now reads: “*Some local governments in China have introduced subsidized HPV vaccination programs for girls aged 13–14, but these exclude catch-up vaccinations for older girls (15–18), leaving low coverage rates (<15%) among this age group*”. (lines 99-101)

In addition, the study setting is described in the Methods section as follows: “*This was a two-arm randomized controlled trial (RCT) conducted in Chengdu, Sichuan Province, China. Chengdu had a population of 21 million people in 2022. Chengdu is* *located in western China, a less developed region with significant economic disparities, and itself exhibits uneven development, containing both affluent and underdeveloped areas. The trial was organized at four community health centers that provide routine vaccination services.*” (lines 135-139)

**Comment 29:**

Line 342: As no information is available on baseline vaccine confidence, it is not possible to claim that the intervention was associated to higher confidence. Also, the costs are not comparable. Please revise.

**Response:**

We appreciated the feedback. With an RCT design, we were relatively confident about the association between the intervention and outcomes, but we agree that it is not appropriate to conclude a causal relationship which should be measured using pre- and post-intervention design. We have revised the sentence to tone down: “*Participants in the intervention arm showed greater vaccine confidence, less vaccine delay, and generated community messages and engagement compared to those in the control arm*”. (lines 360-362)

**Comment 30:**

Please provide arguments in the introduction for the qualification of the study setting as « middle-income country ».

**Response:**

We have added justification for classifying the study setting as a middle-income country in the background section. It now reads: “*Public engagement is essential for tackling such barriers and gaining community acceptance of HPV vaccination [5, 6], but effective community-engaged programs are limited [7] Most evaluations of HPV interventions have been conducted in high-income countries, with relatively few studies carried out in middle-income countries [8]*.” (lines 95-98)

**Comment 31:**

Please discuss in how far the trial participants represent the population of the town and selected health centers.

**Response:**

Unfortunately, public population-level characteristics data are not available. We are therefore unable to compare between our project participants and the general population in the region. In terms of the sampling process, the study sites were selected based on the availability of vaccines, organizational willingness, and capacity to collaborate. We considered the possibility of selection bias brought by this sampling method and used a simple random sampling method to select individual participants to minimize biases at the community level. For a trial to test the *efficacy* of a novel behavioral intervention strategy, we tried to increase the internal validity using random selection of participants and random assignment of intervention. But for our ongoing scale-up *effectiveness* trial, we are collaborating with local CDCs and governments to improve population representativeness.

We acknowledged in the discussion section that “*Selection bias caused by convenience sampling of study sites is possible, and generalizability to the general population should be made cautiously*.” (lines 422-423)

**Comment 32:**

Given the fact that actual donations were of small amount compared to the vaccine prices and of what was announced as community-subsidy, is this really a « pay-it-forward » strategy? Isn't is ethically worrisome to give wrong information about the subsidy?

**Response:**

Sorry about the confusion the wording caused. The main purpose of this project is to use an innovative pro-social strategy to motivate vaccination, while community donations and handwritten postcards are fully voluntary and these are additional benefits. We revised the wording to “community-supported subsidy” to highlight community generosity and kindness. The Chinese wording of pay-it-forward we used during the project implementation reads “爱心接力种” (meaning passing on the kindness and getting vaccinated).

**Comment 33:**

As the authors point out more analyses (qualitative?) are needed to understand which element of the intervention had the positive impact. As it is, one cannot conclude that it is specifically the donation - it may just be the messages. Overall, this should be better captured by all parts of the manuscript, including the title (rather speak about stimulation of pro-social behaviour?).

**Response:**

We agree that the pay-it-forward strategy is a multi-component intervention and it is not possible to isolate the effect of each individual component. To align with the journal requirement on the manuscript title, we decided to keep the current title so that key elements and focus of the project can be well reflected. We added this limitation in the paper “*Third, the pay-it-forward strategy is a multi-component intervention, and its individual effects cannot be isolated. Future research should consider designs that allow disentanglement of individual components*”. (lines 417-420)

**Comment 34:**

Please discuss whether this call for pro-social behaviour could have stimulated reactance, ie, it could have annoyed some caregivers who felt urged into vaccination. Did you conduct any evaluation of user satisfaction?

**Response:**

Thank you for raising this concern. We did not observe or record reactance from our participants during the study implementation. We assessed satisfaction in our earlier feasibility pilot study, which demonstrated that the intervention was acceptable and well-received by participants (please find the publication below), but we did not collect user satisfaction data for the current trial due to concerns about questionnaire length.

Ref: Li Y, Qin C, Li KT, He Y, Qiu S, Wu D, et al., "Feasibility and acceptability of pay-it-forward in increasing uptake of hpv vaccination among 15-18-year-old girls in china: Pilot rct results," Cancer Prevention Research (2025): <http://dx.doi.org/10.1158/1940-6207.CAPR-24-0549>

**Comment 35:**

Line 350: Confusing: how can the follow-up vaccine uptake in the intervention arm be compared to national vaccine coverage data? Please revise this. Please avoid the term « vaccination rate », as it is ambiguous between coverage and uptake incidence among unvaccinated.

**Response:**

The primary outcome of our study was to compare HPV vaccine uptake between the intervention and control arms. We mentioned the national vaccine coverage mainly to contextualize our interpretation of the results. We have replaced the ambiguous term “vaccination rate” with “*HPV vaccine uptake*” throughout the manuscript for clarity.

**Comment 36:**

Line 354: Calling a study « old » is a bit clumsy? Please make it clear whether the UK study addressed a catch-up programme, as well.

**Response:**

We have revised the phrase “old” to “*somewhat dated*”. (lines 373)

Additionally, we clarified that the UK study targeted a catch-up population, specifically “*girls aged 16–18 years*”. (lines 372)

**Comment 37:**

Discuss the contradiction between stratified results by household income and center D.

**Response:**

Thank you. Stratified results showed higher HPV vaccine uptake among lower-income households, but Center D—located in the least developed area—showed no improvement. There are two main reasons for this seemingly contradiction. First, the economic levels of study sites were assessed by using GDP per capita released by the government. Participants clasped by income levels (individual-level measurement) might not necessarily equal to the government reported economic levels (population-level measurement). Second, we speculate that participants in the least developed region might be more affected by poor awareness of HPV vaccine, rather than financial capacity, but more research is warranted to test this assumption.

**Comment 38:**

Line 371: Please revise the statement about the replacement of public investment by pay-if-forward. If this is this the authors' wish, then it should be formulated as such.

**Response:**

We have revised the sentence for clarity. It now reads: “*Of note, pay-it-forward is not intended to replace the government role, but rather to serve as an incentive structure or complementary model to support future catch-up vaccination programs.*” (lines 392-394)

## Response to reviewer 3

**Comment 1:**

Helpful and insightful research. A few queries- in Background - lines 84-87- do parents know if wait on HPV vaccine that they will have to pay ie gov't not offer HPV vaccine free to those 15yr and over? Do they know how much it costs?

**Response:**

Thank you. Since our project targeted girls aged 15 or older, and our participants are not eligible for the government-funded HPV vaccination programs which support girls aged 13-14 years only. Most participants were unaware of the vaccine cost before the trial, but we provided them with the market prices of different HPV vaccines.

**Comment 2:**

The overview of the pay it forward model paper and appendix has one other aspect not noted in text - not in lines 89 thru 95 nor in Figure 1 in supplemental information. Appendix page 10 and 11 notes recruitment phone call told " Your girl is eligible for HPV vaccination through our project and she is lucky to be selected out of >1000 girls living in the neighborhood". ie have won a lottery chance -While equal for both arms RCT but merits some comment in paper to note that this may also have been a nudge to get HPV vaccine - "chance to win a prize" makes someone feel special and more positive- another kind of nudge- here prize is 50% opportunity for subsidized vaccine not true standard of care where no chance to win subsidy.

**Response:**

Sorry about the confusion and thank you for the kind suggestion. We agree that this might have been an additional nudge to get HPV vaccine, unfortunately the design does not allow us to draw this conclusion. The recruitment phone call, which framed participation as a “lottery-like” opportunity, was used mainly to motivate caregivers to visit our clinics and participate in the study and participants in both arms received the same messages during the telephone recruitment, ie, each participant had a 50% chance to be assigned to either pay-it-forward community-supported subsidy or control. After careful considerations, we decided to not add this point to the discussion to not overclaim the effects of a component that is not scientifically tested in this study.

**Comment 3:**

This point could be raised in the discussion . Recruitment occurred over 8 months - was there any difference in volunteer/spontaneous  non study uptake of HPV vaccine in that time period?

**Response:**

Thank you for raising this point. Recruitment spanned eight months due to interruptions from COVID-19. Both intervention and control arms were conducted in parallel, so any time-related effects would be balanced. There were no government-led catch-up programs for HPV vaccination during the study implementation period, and we believe non-study uptake was minimal. We have noted this in the manuscript as a limitation: “*Second, our study had a clinic attendance rate of 54% and a long recruitment period.*” (lines 413)

**Comment 4:**

Outcomes section - as noted above - " standard of care arm" was not really standard of care ie received a phone call and told in lottery to win subsidized vaccine- need another term.

**Response:**

Thank you. Please kindly refer to our response to the second comment you raised. And to clarify reasons for a higher uptake in the control arm than previously reported uptake rate of 8.6%, we added the following information in the discussion: “*Additionally, our control arm demonstrated higher uptake (17.5%) compared to the previously reported rate of 8.6% in western China. This difference may be attributable to implementation components such as telephone recruitment reminders, educational pamphlets addressing knowledge gaps, and established trust in community health centers.”* (lines 379-382)

We also revised the “standard-of-care” arm to “control” arm for accuracy throughout the manuscript.

**Comment 5:**

Costs - lines 227 -228- in supplemental materials page 11 & 12 not clear if telephone costs included personnel costs to make the phone call as well as telephone service charges.

**Response:**

Thank you for your comment. We would like to clarify that the telephone cost reported in the Table A in S1 File refers only to airtime charges, specifically the cost billed by the mobile network provider at a rate of 0.15 yuan per minute. These charges do not include personnel time or labor costs associated with making the calls. We have updated the supplemental materials to make this distinction more explicit.

**Comment 6:**

Results - not clear if most caregivers knew cost of HPV vaccine before trial- did they? , was that asked?

**Response:**

We did not assess caregivers’ baseline awareness of vaccine costs before they received our intervention materials. We provided participants in both arms with market prices of different vaccines via the educational pamphlet. We have clarified this in the Methods section: “*Caregiver-girl pairs in the control arm received a routine educational pamphlet used in clinics which included the prices of available HPV vaccines.*” (lines 187-188)

**Comment 7:**

Discussion- again in line 341 refer to standard of care when these also received a phone call and a chance at a lottery to win discounted cost of vaccine - not standard of care - and furthermore the rate of vaccine uptake was 17.5% (line 286) and this is over 2x rate reported for this age group in " real" standard of care - 8.6%-

**Response:**

Thank you for this observation. We acknowledge that the standard-of-care arm was not equivalent to actual routine care and that uptake was higher (17.5% vs. 8.6%). There may be several reasons. First, as the reviewer suggested, the telephone recruitment from the community health center might serve as a reminder to our participants. Second, the education pamphlet might have provided useful information to improve awareness and knowledge. Third, delivering the intervention in community health centers as part of the health services might have increased caregiver trust and acceptance of HPV vaccines. We have added the above information in the discussion section which reads as “*Additionally, our control arm demonstrated higher uptake (17.5%) compared to the previously reported rate of 8.6% in western China. This difference may be attributable to implementation components such as telephone recruitment reminders, educational pamphlets addressing knowledge gaps, and established trust in community health centers.”* (lines 379-382)

**Comment 8:**

line 102. This is a great increase albeit not sure how much cost for those phone calls beyond services costs lists and clinic time to learn not won lottery and given pamphlet. Looks like big bang for small money. Other reminder call studies have shown good impact. This is modified reminder. So yes the pay it forward did even more than this other control arm but that arm. These gains in the control arm deserve some attention too. This is where the " standard of care " issue could be raised as noted above - really control arm and could describe how differs for standard of care. This bonus uptake in control arm deserves attention in the discussion.

**Response:**

Agreed. We have added these discussions “*Additionally, our control arm demonstrated higher uptake (17.5%) compared to the previously reported rate of 8.6% in western China. This difference may be attributable to implementation components such as telephone recruitment reminders, educational pamphlets addressing knowledge gaps, and established trust in community health centers*.” (lines 379-382)

## Response to editorial

**Comment 1:**

Please upload any figures associated with your paper as individual TIF or EPS files with 300dpi resolution at resubmission; please read our figure guidelines for more information on our requirements: http://journals.plos.org/plosmedicine/s/figures. While revising your submission, please upload your figure files to the PACE digital diagnostic tool, https://pacev2.apexcovantage.com/. PACE helps ensure that figures meet PLOS requirements. To use PACE, you must first register as a user. Then, login and navigate to the UPLOAD tab, where you will find detailed instructions on how to use the tool. If you encounter any issues or have any questions when using PACE, please email us at PLOSMedicine@plos.org.

**Response:**

We have uploaded all figures as individual TIF file, in accordance with PLOS Medicine’s figure guidelines

**Comment 2:**

We expect all researchers with submissions to PLOS in which author-generated code underpins the findings in the manuscript to make all author-generated code available without restrictions upon publication of the work. In cases where code is central to the manuscript, we may require the code to be made available as a condition of publication. Authors are responsible for ensuring that the code is reusable and well documented. Please make any custom code available, either as part of your data deposition or as a supplementary file. Please add a sentence to your data availability statement regarding any code used in the study, e.g. "The code used in the analysis is available from Github [URL] and archived in Zenodo [DOI link]" Please review our guidelines at https://journals.plos.org/plosmedicine/s/materials-software-and-code-sharing and ensure that your code is shared in a way that follows best practice and facilitates reproducibility and reuse. Because Github depositions can be readily changed or deleted, we encourage you to make a permanent DOI'd copy (e.g. in Zenodo) and provide the URL.

**Response:**

All custom code used in this study has been made available on Appendix D in S1 File to ensure permanent access and reproducibility.

**Comment 3:**

Please ensure that the study is reported according to the CONSORT guideline and include the completed CONSORT checklist as Supporting Information. When completing the checklist, please use section and paragraph numbers, rather than page numbers. Please add the following statement, or similar, to the Methods: "This study is reported as per CONSORT guideline (S1 Checklist)."

**Response:**

We have completed the CONSORT checklist, using section and line numbers as requested.

**Comment 4:**

Abstract: Please structure your abstract using the PLOS Medicine headings (Background, Methods and Findings, Conclusions). In the last sentence of the Abstract Methods and Findings section, please describe the main limitation(s) of the study's methodology.

**Response:**

We have also added a sentence at the end of the “Methods and Findings” section of the abstract to describe the main methodological limitation of the study. It reads: *“The trial had several limitations, including a 54% clinic attendance rate, and its conduct in a single western province.”* (lines 56-57)

**Comment 6:**

At this stage, we ask that you include a short, non-technical Author Summary of your research to make findings accessible to a wide audience that includes both scientists and non-scientists. The Author Summary should immediately follow the Abstract in your revised manuscript. This text is subject to editorial change and should be distinct from the scientific abstract. Ideally each sub-heading should contain 2-3 single sentence, concise bullet points containing the most salient points from your study. In the final bullet point of 'What Do These Findings Mean?', please include the main limitations of the study in non-technical language. Please see our author guidelines for more information: <https://journals.plos.org/plosmedicine/s/revising-your-manuscript#loc-author-summary>.

**Response:**

We have added Author Summary on Page 4-5.

**Comment 7:**

Please express the main results with 95% CIs as well as p values. When reporting p values please report as p<0.001 and where higher as the exact p value p=0.002, for example. Throughout, suggest reporting statistical information as follows to improve clarity for the reader "22% (95% CI [13%,28%]; p</=)". Please be sure to define all numerical values at first use.

**Response:**

We have revised the format of all percentage confidence intervals accordingly.

**Comment 8:**

Please cite the reference numbers in square brackets. Citations should precede punctuation.

**Response:**

We have revised the manuscript so that citations now precede punctuation throughout, in accordance with journal style.

**Comment 9:**

Please provide titles and legends for all figures and tables (including those in Supporting Information files).

Please define all abbreviations used in each figure/table (including those in Supporting Information files).

**Response:**

We have provided titles and legends for all figures and tables, including those in the Supporting Information files (Page 26-27). Additionally, all abbreviations used in each figure and table have been clearly defined.

**Comment 10:**

Please cite your Supporting Information as outlined here: https://journals.plos.org/plosmedicine/s/supporting-information

**Response:**

We have revised the manuscript to cite all Supporting Information files according to PLOS Medicine’s guidelines.

**Comment 11:**

Please include absolute numbers wherever you report percentages; eg, n/N (%)

**Response:**

We have revised the manuscript to include absolute numbers alongside all percentages, following the format n/N (%), as requested.

**Comment 12:**

Please ensure that the funders and grant numbers match between the Financial Disclosure field and the Funding Information tab in your submission form. Note that the funders must be provided in the same order in both places as well.

Currently, order of grants are different in both places.

2) Please amend your detailed Financial Disclosure statement. This is published with the article, therefore should be completed in full sentences and contain the exact wording you wish to be published.

i). State the initials, alongside each funding source, of each author to receive each grant.

3) Please amend your detailed Financial Disclosure statement. This is published with the article, therefore should be completed in full sentences and contain the exact wording you wish to be published.

i). State what role the funders took in the study. If the funders had no role in your study, please state: “The funders had no role in study design, data collection and analysis, decision to publish, or preparation of the manuscript.”

ii). If any authors received a salary from any of your funders, please state which authors and which funders.

**Response:**

We have revised the Financial Disclosure statement to ensure that the funders and grant numbers match the order listed in the Funding Information tab of the submission system.

**Comment 13:**

6) Some material included in your submission may be copyrighted. According to PLOS’s copyright policy, authors who use figures or other material (e.g., graphics, clipart, maps) from another author or copyright holder must demonstrate or obtain permission to publish this material under the Creative Commons Attribution 4.0 International (CC BY 4.0) License used by PLOS journals. Please closely review the details of PLOS’s copyright requirements here: PLOS Licenses and Copyright. If you need to request permissions from a copyright holder, you may use PLOS's Copyright Content Permission form.

Potential Copyright Issues:

1) [Appendix Figure 4]: Please confirm (a) that you are the photographer; or (b) provide written permission from the photographer to publish the photo(s) under our CC BY 4.0 license.

2) "[Clipart in Appendix Figure 1, Appendix Figure 3, Appendix Figure 4]: Please confirm whether you drew the images / clip-art within the figure panels by hand. If you did not draw the images, please provide (a) a link to the source of the images or icons and their license / terms of use; or (b) written permission from the copyright holder to publish the images or icons under our CC BY 4.0 license. Alternatively, you may replace the images with open source alternatives. See these open source resources you may use to replace images / clip-art:

- https://commons.wikimedia.org

- <https://openclipart.org/>"

**Response:**

We have obtained written copyright permission for Appendix Figure 4 (Fig D in S1 File), which was created by Xiamen Innovax Biotechnology Co., Ltd., to publish the figure under the CC BY 4.0 license.

Appendix Figures 1 and 3 (Fig A and C in S1 File) were created using Canva. According to Canva’s licensing agreement (<https://www.canva.cn/policies/free-media/>), the free media elements used in these figures are permitted for both commercial and non-commercial use, including publication under open-access licenses such as CC BY 4.0.
