# Supplementary material for: Effect of a pay-it-forward strategy on reducing HPV vaccine delay and increasing uptake among 15- to 18-year-old girls in China: A randomized controlled trial
Source: PLoS Med. 2025 Jul 31;22(7):e1004535. doi: 10.1371/journal.pmed.1004535 (PMC12331080; doi:10.1371/journal.pmed.1004535)
Supplement: Attachment — Submitted filename: final_R2_author response_PlosMed_24June2025.docx [file pmed.1004535.s009.docx]

# Author response

## Response to editors

**Comment 1**

*Please change your title, to be non-declarative.

* Please confirm that your title complies with to PLOS Medicine's style. Your title must be nondeclarative and not a question. It should begin with main concept if possible. "Effect of" should be used only if causality can be inferred, i.e., for an RCT. Please place the study design ("A randomized controlled trial," "A retrospective study," "A modelling study," etc.) in the subtitle (ie, after a colon).

**Response:**

Thank you for the suggestion. We have revised the title to align with PLOS Medicine’s style guidelines. The new title is: “Effect of pay‑it‑forward strategy to reduce HPV vaccine delay and increase uptake among catch-up age girls: A randomized controlled trial” (Lines 1–2)

**Comment 2**

*please specify what '54% clinic attendance rate refers to.

**Response:**

Thank you for the comment. We have clarified this as follows: “The trial had several limitations, including a 54% clinic attendance rate (360 of 662 consented participants attended) and its conduct in a single western province of China.” (lines 63-65)

**Comment 3**

*Please provide details about randomisation

**Response:**

We have updated the abstract to describe the randomization process: “Eligible participants were unvaccinated girls living in the service areas of CHCs, and were randomly assigned using the sealed envelope method to either the pay-it-forward arm (received a community subsidy of 329 RMB and an opportunity to support others) or control arm (self-paid vaccination at the market price).” (lines 48-51)

**Comment 4**

* Please provide details about study setting

**Response:**

We have included the study setting in the abstract: “This study was conducted from July 6, 2022, to June 9, 2023, in four community health centers (CHCs) in Chengdu, western China.” (lines 47-48)

**Comment 5**

* Please specify who was blinded to the intervention and control.

**Response:**

We have clarified the blinding procedure: “Participants were unblinded only after the envelope was opened, while the CHC staff coordinators, physicians prescribing the vaccine, outcome assessors, and data analysts were blinded to the intervention allocation.” (lines 52-54)

**Comment 6**

* Please state that analysis was intention to treat.

**Response:**

We have added this information to the abstract: “Data were analyzed using the intention-to-treat approach” in the abstract. (lines 55-56)

**Comment 7**

* Please provide the number of participants lost to follow up in each group.

**Response:**

We have added the following to the abstract: “A total of 321 participants were enrolled and none were lost to follow-up during the 3-month post-intervention period” in the abstract. (lines 56-57)

**Comment 8**

* Please indicates the dates during which study enrollment and follow up occurred.

**Response:**

We have clarified this in the abstract: “This study was conducted from July 6, 2022, to June 9, 2023, in four community health centers (CHCs) in Chengdu, western China” in the abstract. (lines 47-48)

**Comment 9**

* Please clarify that the prices that are mentioned for the vaccine in both arms were not actually what was paid. Please ensure this is clear throughout the manuscript.

**Response:**

We have clarified this point in the abstract and throughout the manuscript. The abstract now reads: “Eligible participants were unvaccinated girls living in the service areas of CHCs, and were randomly assigned using the sealed envelope method to either the pay-it-forward arm (received a community subsidy of 329 RMB and an opportunity to support others) or control arm (self-paid vaccination at the market price).” (lines 48-51)

**Comment 10**

* Please include 'Chinese' in the last sentence of the Methods and Findings paragraph (in “conduct in a single western province”)

**Response:**

Thank you for your suggestion. We have revised the sentence to clarify the geographic setting as follows: “The trial had several limitations, including a 54% clinic attendance rate (360 of 662 consented participants attended) and its conduct in a single western province of China.” (lines 63-65)

**Comment 11**

Statements:

*please remove funding statement from the main text (in the Method sections)

*Please remove the funding statement from the main text, at the end of the discussion (statement starting on line 442).

*please include a statement on code in DAS.

* The funding statement should include: specific grant numbers, initials of authors who received each award, URLs to sponsors’ websites. Also, please state whether any sponsors or funders (other than the named authors) played any role in study design, data collection and analysis, the decision to publish, or preparation of the manuscript. If they had no role in the research, include this sentence: “The funders had no role in study design, data collection and analysis, decision to publish, or preparation of the manuscript.

**Response:**

Thank you. We have removed the funding statement from the Methods and Discussion sections of the main text and have moved it to the appropriate funding section. The updated statement reads: “The Bill & Melinda Gates Foundation (INV-034554, INV-003174 to DW, JL and ST), National Natural Science Foundation of China (82473742 to DW), US NIH (NIAID R01AI158826 to JDT), Nanjing Medical University Career Development Grant (NMUR20230008 to DW), Jiangsu Provincial Professorship Career Development Grant (KY103R202309 to DW) funded this study. The study protocol underwent review by independent reviewers appointed by the funder prior to grant allocation. The funders had no role in study design, data collection and analysis, decision to publish, or preparation of the manuscript. The authors are fully responsible for the content of this manuscript, and the views and opinions described in the publication reflect solely those of the authors.” (lines 457-465)

We have added the code statement in the data availability which now reads “Anonymous data with code book and data analysis code used in the main analysis are available in the Supporting Information files (Appendix C and Appendix D in S1 File).” (lines 485-486)

**Comment 12**

Checklist:

*as your study was affected by COVID, please fill out the CONSERVE-CONSORT checklist.

*Please ensure that the checklists mentions paragraphs and sections rather than line numbers.

**Response:**

Thank you. We have completed the CONSERVE-CONSORT checklist and revised the CONSORT checklist accordingly. All references now point to paragraph numbers and section titles instead of line numbers, in line with journal requirements.

**Comment 13**

* Please confirm that your abstract complies with our requirements, including format (three sections: Background, Methods and Findings, and Conclusions) and providing all the information relevant to this study type https://journals.plos.org/plosmedicine/s/submission-guidelines#loc-abstract

**Response:**

We confirm that the abstract adheres to the required format, including three clearly labeled sections—Background, Methods and Findings, and Conclusions—and includes all required elements for randomized controlled trials as per PLOS Medicine’s submission guidelines.

**Comment 14**

* Please ensure that all abbreviations are defined at first use throughout the text.

**Response:**

We have carefully reviewed the entire manuscript and ensured that all abbreviations are spelled out at first mention in both the main text and supplementary materials.

**Comment 15**

* Please confirm that all numbers presented in the abstract are present and identical to numbers presented in the main manuscript text.

**Response:**

We have thoroughly checked and confirmed that all numerical values in the abstract are consistent with those presented in the main text of the manuscript.

**Comment 16**

* In the author summary, in the final bullet point of 'What Do These Findings Mean?', please include the main limitations of the study in non-technical language.

**Response:**

Thank you for the suggestion. We have revised the final bullet point in the ‘What Do These Findings Mean?’ section to include key limitations in plain language: “However, this study was conducted in one province in western China.” **(line 91)**

**Comment 17**

* All authors must declare their relevant competing interests per the PLOS policy, which can be seen here: https://journals.plos.org/plosmedicine/s/competing-interests For authors with ties to industry, please indicate whether any of the interests has a financial stake in the results of the current study.

* Please add this statement to the manuscript's Competing Interests: "EHG is an Academic Editor on PLOS Medicine's editorial board.

**Response:**

We have added the “I have read the journal's policy and the authors of this manuscript have the following competing interests: EHG is an Academic Editor on PLOS Medicine's editorial board.” in the declaration of interests. All other authors have declared no competing interests related to this study.

**Comment 18**

*We appreciate that you provide all the data in an Excel file. However, it appears that you have included data that may breach patient confidentiality. Please edit your data set in accordance with patient consent, and remove the following personal data: birthdates

*Please also labels all columns in this excel file properly, so the file is understandable for readers.

**Response:**

Thank you for highlighting this issue. We have removed all personally identifiable data, including birthdates, from the dataset to maintain participant confidentiality. We have also ensured that all columns in the dataset are clearly labeled and correspond to the codebook provided in Appendix C of the S1 File, to facilitate understanding by readers.

**Comment 19**

Potential Copyright Issues: Supplementary Appendix figure A, B, C: Please confirm whether you drew the images / clip-art within the figure panels by hand. If you did not draw the images, please provide (a) a link to the source of the images or icons and their license / terms of use; or (b) written permission from the copyright holder to publish the images or icons under our CC BY 4.0 license. Alternatively, you may replace the images with open source alternatives. See these open source resources you may use to replace images / clip-art:

- https://commons.wikimedia.org

- https://openclipart.org/.

**Response:**

Thank you for your comment regarding the potential copyright issues.

Appendix Figures A and C (Figures A and C in the S1 File) were created using Canva. In accordance with Canva’s Content License Agreement (https://www.canva.com/policies/content-license-agreement/), the free media elements used in these figures are permitted for both commercial and non-commercial use, including redistribution and modification under open-access licenses such as CC BY 4.0.

Figure B in S1 File was created entirely using Microsoft PowerPoint, with all graphic elements (shapes, lines, and layout) drawn and designed manually by the authors. No third-party or copyrighted images were used in this figure.

For Figure D, we have obtained the necessary copyright permission and have included the authorization letter in the Supporting Information as requested.

## Response to reviewer 1

**Comment 1**

Figure 2 is a good representation of the sub-group analysis, but the reporting of this in the methods needs revision. The interaction p-value is what provides evidence for heterogeneity - in this case it was only awareness of HPV vaccinations which had heterogeneity. Making inferences about when sub-groups meets significance (or had a confidence limit that crosses the null threshold) is called 'differences in nominal significance' and is creates a liberal bias in inference, e.g. in many of these subgroups the CL cross. For this section, the main result is just for awareness of HPV vaccinations, with none of the rest of the subgroups showing evidence of heterogeneity.

**Response:**

Thank you for pointing this out. We have revised the Methods section to clarify that interaction terms were used to assess effect modification. The revised sentence now reads: “To test for heterogeneity of the intervention effect across subgroups, we included interaction terms between the intervention group and each subgroup variable in the regression models.” (lines 293-294)

## Response to reviewer 2

**Comment 1:**

My preference would be that your clear answers would lead to some clarification in the manuscript on ALL points - assuming that if one aspect was not clear to me, it may not be clear to readers, either. This concerns questions 9, 10, 13, 22, 24, 34 and 37.

**Response:**

Thank you for your helpful suggestion. We have revised the manuscript to incorporate clarifications for questions 9, 10, 13, 22, 24, 34, and 37 to ensure clarity for future readers. The corresponding changes are as follows:

- Comment 9:

We added: “Donations were integrated into the program’s operational budget and used to support future HPV vaccinations for eligible girls in the same community.” (lines 204-205)

- Comment 10

We added: “Despite small mean donations collected through the pay-it-forward strategy, we observed higher average donations in the two better-off regions than in the lower-income regions, suggesting the potential of creating a subsidization mechanism to support HPV vaccination in poorer regions.” (lines 403-406)

- Comment 13

We added: “Telephone recruitment included a brief introduction to the overall study purpose, procedures, and associated benefits and risks (Appendix A in S1 File).” (lines 171-172).

We also revised: “Participating caregivers provided verbal consent during telephone recruitment and signed a written informed consent form, and girls provided assent during the on-site visit.” (lines 176-178)

- Comment 22

We clarified in the methods: “Similarly, comparisons of these secondary outcomes between the two arms were also assessed using generalized linear models with Gaussian distribution and identity link function.” (lines 273-275)

- Comment 24

We added: “Additional studies are also needed to assess the feasibility of integrating these solutions into routine services.” (lines 454-455)

- Comment 34

We added “Our earlier pilot trial demonstrated the intervention process was acceptable and well-received by participant [29].” (lines 225-226)

- Comment 37

We added: “However, Center D—located in the least developed area—showed no improvement. This may be because limited awareness of the HPV vaccine, rather than financial constraints, was the primary barrier in this region, though further research is needed to confirm this.” (lines 390-392)

***Original Comments Referred to in Comment 1:***

Comment 9: Please explain what the donations were being used for and in which way. Eg, are they given to specific users (according to which rules?), or integrated in the health center budget? In absence of this information - and given the eventually small donations made (relative to the vaccine price and the subsidy claim) -, it is not clear whether this can really be called a pay-it-forward strategy.

Response: Thank you for the important question. Donations were integrated into the program’s operational budget and used to support future HPV vaccinations for eligible girls in the same community. While individual donations may be small relative to the vaccine cost, they serve as a key component of the prosocial mechanism—highlighting voluntary generosity and fostering a sense of collective support. The primary goal of using the model in this project is to promote vaccine uptake through kindness and altruism(ref). Ref: Byrne M, Tan RKJ, Wu D, Marley G, Hlatshwako TG, Tao Y, et al., "Prosocial interventions and health outcomes: A systematic review and meta-analysis," JAMA Netw Open 6 (2023):e2346789-e2346789, <http://dx.doi.org/10.1001/jamanetworkopen.2023.46789>.

Comment 10: Another point is that social inequalities are not at all addressed, although financial barriers are presented as essential and part of the lever of the evaluated strategy. Please present a few data from China.

Response: We agree this is an important issue. Based on our data (Table 2), participants from Sites A and B—more economically developed areas—had higher donation rates and contributed larger amounts. This suggests the potential of the pay-it-forward strategy to enable individuals in better-off areas to support those in underserved communities. Although this strategy may not directly reduce systemic social inequalities, it holds promise in promoting solidarity and facilitating access to vaccination through community solidarity.

Comment 13: Line 135: How was the purpose of the visit at the center presented to the families? Any notion of HPV, of a randomised study? Where and when did caregivers provide consent, before or during the visit?

Response: The purpose of the study and the visit at the center was explained during the telephone recruitment, and the telephone recruitment script is available in the Appendix A in S1 File. During the telephone recruitment process, participants were given a brief introduction to HPV and cervical cancer, and were informed about the 50% chance of being assigned to the pay-it-forward arm or the control arm.

We revised the sentence in the manuscript: “Participating caregivers provided verbal consent during telephone recruitment and signed a written informed consent form, and girls provided assent during the on-site visit.” (lines 166-168).

Comment 22: How were vaccine confidence data analysed? Please conduct effectiveness analyses stratified by vaccine confidence levels.

Response: Thank you for your suggestion. We did not collect vaccine confidence data at baseline; therefore, it is not possible to conduct stratified effectiveness analyses by baseline confidence. We recognize this as a limitation. However, individual randomization helps balance potential confounders, including vaccine confidence, between arms. Vaccine confidence was assessed as a secondary outcome during follow-up.

Comment 24: Also, which percentage of the no-shows were vaccinated? If all, then the 48% los should be presented as a major barrier of the intervention feasibility. Please discuss whether the intervention can be integrated in other center visits (routine or for specific health issues).

Response: Unfortunately, we were not able to collect information about their vaccination status without their consent to participate in the study. We agree that solutions to improve participants’ willingness to participate in health projects and access vaccination services will be important. Our group are currently conducting research to explore the feasibility of integrating these solutions into routine clinic and school-based vaccination programs via collaborating with local CDCs and community health centers. We hope to share more insights in the near future.

Comment 34: Please discuss whether this call for pro-social behaviour could have stimulated reactance, ie, it could have annoyed some caregivers who felt urged into vaccination. Did you conduct any evaluation of user satisfaction?

Response: Thank you for raising this concern. We did not observe or record reactance from our participants during the study implementation. We assessed satisfaction in our earlier feasibility pilot study, which demonstrated that the intervention was acceptable and well-received by participants (please find the publication below), but we did not collect user satisfaction data for the current trial due to concerns about questionnaire length. Ref: Li Y, Qin C, Li KT, He Y, Qiu S, Wu D, et al., "Feasibility and acceptability of pay-it-forward in increasing uptake of hpv vaccination among 15-18-year-old girls in china: Pilot rct results," Cancer Prevention Research (2025): <http://dx.doi.org/10.1158/1940-6207.CAPR-24-0549>

Comment 37: Discuss the contradiction between stratified results by household income and center D.

Response: Thank you. Stratified results showed higher HPV vaccine uptake among lower-income households, but Center D—located in the least developed area—showed no improvement. There are two main reasons for this seemingly contradiction. First, the economic levels of study sites were assessed by using GDP per capita released by the government. Participants clasped by income levels (individual-level measurement) might not necessarily equal to the government reported economic levels (population-level measurement). Second, we speculate that participants in the least developed region might be more affected by poor awareness of HPV vaccine, rather than financial capacity, but more research is warranted to test this assumption.

**Comment 2:**

For Question 31, I rather was thinking of external validity of the trial results, not of selection bias. Your trial results are certainly internally valid, indepent of the selection of participants. However, they may not be applicable to any resident, if participants were highly selected.

**Response:**

Thank you for this important clarification. We agree that while our trial demonstrates strong internal validity, its external validity depends on how representative our participants are of the broader population. Although not all individuals invited agreed to participate, our sample was drawn using random sampling rather than purposive sampling. Therefore, participants were not highly self-selected, and the characteristics of those enrolled are still likely to be broadly representative of the target population in the service areas. This enhances the external validity of our findings.
