# Supplementary material for: Effect of a pay-it-forward strategy on reducing HPV vaccine delay and increasing uptake among 15- to 18-year-old girls in China: A randomized controlled trial
Source: PLoS Med. 2025 Jul 31;22(7):e1004535. doi: 10.1371/journal.pmed.1004535 (PMC12331080; doi:10.1371/journal.pmed.1004535)
Supplement: Attachment — Submitted filename: final_R3_author response_PlosMed_15July2025.docx [file pmed.1004535.s010.docx]

# Author response

**Comment 1:**

We suggest to change your title to “Effect of a pay-it-forward strategy on reducing HPV vaccine delay and increasing uptake among 15- to 18-year-old girls in China: A randomized controlled trial”.

**Response:**

We have revised the title as suggested: “Effect of a pay-it-forward strategy on reducing HPV vaccine delay and increasing uptake among 15- to 18-year-old girls in China: A randomized controlled trial”.

**Comment 2:**

Please include URLs in the funding statement.

**Response:**

We have added the URLs as requested. The funding statement now reads: “The Bill & Melinda Gates Foundation (INV-034554, INV-003174 to DW, JL and ST) (https://www.gatesfoundation.org/), National Natural Science Foundation of China (82473742 to DW) (https://www.nsfc.gov.cn/english/site_1/index.html), US NIH (NIAID R01AI158826 to JDT) (https://www.nih.gov/), Nanjing Medical University Career Development Grant (NMUR20230008 to DW) (https://english.njmu.edu.cn/), Jiangsu Provincial Professorship Career Development Grant (KY103R202309 to DW) (https://www.jiangsu.gov.cn/art/2022/1/4/art_46144_10283684.html) funded this study.” (lines 466-472)

**Comment 3:**

Please mention the CONSERVE-CONSORT checklist in the main text (with a reference to the checklist), and state why you completed it (e.g. study affected by the pandemic).

**Response:**

Thanks for your suggestion. We have mentioned the CONSERVE-CONSORT checklist in Methods, which now reads “We used CONSORT guidelines [27] and CONSERVE (for this trial affected by the COVID-19 pandemic) [28] for reporting this RCT trial (S1 Checklist and S2 Checklist).” (lines 167-169) CONSERVE-CONSORT checklist is named S2 Checklist.

Reference 28: Orkin AM, Gill PJ, Ghersi D, Campbell L, Sugarman J, Emsley R, et al. Guidelines for Reporting Trial Protocols and Completed Trials Modified Due to the COVID-19 Pandemic and Other Extenuating Circumstances: The CONSERVE 2021 Statement. Jama. 2021;326(3):257-65.

**Comment 4:**

Please define HPV the first time you mention it in the abstract and the main text.

**Response:**

We have added the definition “human papillomavirus (HPV)” at first mention in both abstract and main text. (lines 44 and 103)

**Comment 5:**

The excel file still contains IDs; please remove these. Please also consider providing a second tab with more information; e.g. what 0 and 1 for arm means.

**Response:**

We have removed all participant IDs from the Excel file and added a supplementary tab labeled “codebook” that explains the variable items and their corresponding value meanings.

**Comment 6:**

Thank you for including a code statement in the Data Availability Statement (DAS). Please ensure that the DAS in your online submission is identical to the one in the manuscript itself.

**Response:**

Thank you for your note. We confirm that the Data Availability Statement in our online submission matches exactly with the version in the manuscript.

**Comment 7:**

Please clearly state that the first vaccine was for free for the intervention arm (rather than stating subsidized, or giving a RMB number.)

**Response:**

We have clarified the subsidy details in the intervention arm. The revised text now states: “caregiver-girl pairs in the pay-it-forward arm received the intervention that included community co-created postcard messages, featuring messages from previous participants on the back (Fig C in S1 File), subsidized HPV vaccination (covering RMB 329, equivalent to 47.70 USD), and the opportunity to voluntarily donate towards someone else’s vaccine dose and/or write postcard messages.” (lines 209-213)

**Comment 8:**

Please use only $ or RMB in the abstract, not both. We are happy if you want to mention both in the methods section.

**Response:**

Thank you for this suggestion. We have revised the manuscript to use only USD ($) in the abstract.

**Comment 9:**

Abstract: thank you for including the data we ask for. However, it now reads slightly confusing. We suggest you rewrite so that the flow goes:

1) We identified 662 participants per phone invitation

2) only 360 showed up in the health centra (this is the number lost to follow up)

3) they then were assigned to experimental or control arm, and unblinded.

4) treatment was ‘pay-it-forward’ or control, primary outcome was first HPV vaccine. Abstract: Consider adding a second sentence to the Conclusions, for instance that the participants in the pay-it-forward method also showed higher vaccine confidence.

**Response:**

We have reorganized as suggested: “Participants were initially recruited via telephone and, after providing verbal consent, attended in-person visit where they were randomly assigned using the sealed envelope method to either the pay-it-forward arm (received a community subsidy of 47.7 USD and an opportunity to support others) or control arm (self-paid vaccination at the market price)”, “We identified 662 participants per phone invitation. A total of 321 participants showed up in the health centers and randomly assigned to the pay-it-forward arm (n=161) or control arm (n=160).” (lines 52-56 and lines 60-62)

**Comment 10:**

Consider adding a second sentence to the Conclusions, for instance that the participants in the pay-it-forward method also showed higher vaccine confidence.

**Response:**

Thank you for the suggestion. We revised the Conclusions as “The pro-social pay-it-forward strategy was effective to increase catch-up HPV vaccination among teenage girls. This approach also enhanced vaccine confidence among participants. Pay-it-forward demonstrates promise as an effective intervention to improve vaccine uptake through community engagement.” (lines 72-75)

**Comment 11:**

We do not publish any copyright or trademark symbols that usually accompany proprietary names, eg ©, ®, ™ (e.g. next to drug or reagent names). Please remove all instances of trademark/copyright symbols throughout the text, including:

® on page 12.

**Response:**

Thank you for bringing this to our attention. We have removed the “®” on page 12 in accordance with your style guidelines.
